# Biodiversity of Northern Italy popcorn: a study on genetic diversity and agronomic performances of traditional landraces

**DOI:** 10.3389/fpls.2025.1536714

**Published:** 2025-06-13

**Authors:** Alessandra Lezzi, Lorenzo Stagnati, Elena Petretto, Giovanna Soffritti, Silvano Lodetti, Graziano Rossi, Alessandra Lanubile, Adriano Marocco, Matteo Busconi

**Affiliations:** ^1^ Dipartimento delle Scienze delle Produzioni Vegetali Sostenibili, DI.PRO.VE.S, Università Cattolica del Sacro Cuore, Piacenza, Italy; ^2^ Centro di Ricerca sulla Biodiversità e sul DNA antico (BioDNA), UniversitàCattolica del Sacro Cuore, Piacenza, Italy; ^3^ Dipartimento di Scienze della Terra e dell’Ambiente, DSTA, Università degli Studi di Pavia, Pavia, Italy

**Keywords:** maize landraces, popcorn, agrobiodiversity, genetic characterization, genetic resources, *in* and *ex situ* conservation

## Abstract

Popcorn (*Zea mays* spp. *mays* - Everta) is an ancient and widely recognized maize type, of American origin, enjoyed for centuries worldwide and still highly valued for its unique popping trait. Italy, considered a secondary center of maize differentiation, still holds a rich diversity of local maize landraces survived on farm and also *ex situ* stored. Despite this genetic wealth, Italian popcorn varieties have largely been neglected in modern breeding programs and remain poorly characterized, with only fragmented and incomplete data available. Recent studies have confirmed the widespread presence of traditional popcorn landraces across Italy, also if relegate to small garden cultivations, yet a comprehensive understanding of their genetic and agronomic traits remains lacking. This underscores the urgent need to preserve and document these landraces to conserve biodiversity and protect Italy’s cultural heritage. In response to this gap, in this study, ten Italian popcorn landraces were collected and morphological characterization was performed. Moreover, a genetic characterization of 282 individuals was conducted using a GBS approach. The morphological characterization revealed significant phenotypic and agronomic variability for a total of 13 traits including susceptibility to fungal infection and popping traits, whereas the genetic one identified 313,342 SNP variants, uncovering evidence of local adaptation and provided insights into population structure by Admixture analysis, which revealed the presence of 8 ancestral populations consistently with morphological and historical data. This work sheds light on these neglected landraces, offering valuable information for biodiversity conservation and future breeding efforts, particularly in marginal areas where smallholder cultivation remains crucial.

## 1 Introduction

Popcorn (*Zea mays* spp. *mays* - Everta) is one of the most loved and well-known maize types worldwide; it has been enjoyed for centuries and holds significant commercial importance today as a ready-to-eat snack product and deserves attention given the increasing demand for its production. The value of the Global Popcorn market was around 3794 million US$ in 2021, which is expected to grow to 5550 million US$ by 2025 with a Compound Annual Growth Rate (CAGR) of 6.7% (Kaur et al., 2021). In Italy the grain production is imported or in any case comes from modern hybrids, without ever having considered the diversity existing at the national level.

The primary characteristic of this maize type is popping traits (expansion) which have been here maximized by human selection (Kaur et al., 2021). It is characterized by hard caryopsis (kernel) with vitreous endosperm in high proportion and an opaque core. Upon heating to a sufficient temperature, the popcorn kernel expands up to 30 times the volume of the original one, producing a characteristic flake (Sweley et al., 2013; Santacruz-Varela et al., 2004). The use of expanded corn kernels is ancient and is among the earliest cereal-based snack foods consumed by humans (Matsuoka et al., 2002), however, limited information about popcorn was written before 1880, when its commercialization began (Sweley et al., 2013; Locatelli et al., 2019). Findings from a study conducted by Matsuoka et al. (2002) strongly support the hypothesis that popcorn is ancient, and it can be found at the very first steps in the domestication history of maize. Some Mexican popcorn varieties were, indeed, found to group immediately after teosinte populations in a phylogram constructed from microsatellite data. This hypothesis has also been proposed by other research groups (Dhawan, 1964; Singh, 1977), which placed popcorn varieties among the oldest ones due to their primitive maize characteristics lacking apical dominance and thus able to tiller, being highly prolific plants (with 5 to 9 ears per stalk) and uniform ear size.

According to Brandolini and Brandolini (2006), Everta-type maize varieties were among the first to be introduced in Italy in the 16th century; a proof of this could be represented by an image reported as number 70 by the famous botanist of the University of Bologna Ulisse Aldrovandi (1522-1605), known as *Maizum seu Triticum Bactrianum*, painted in 1551 (see Brandolini and Brandolini, 2006, pag. 34; https://sma.unibo.it/it/il-sistema-museale/orto-botanico-ed-erbario/collezioni/erbario-lerbario-di-ulisse-aldrovandi).

Popcorn never gained agricultural interest in Italy, remaining confined to family gardens as comfort food (Brandolini and Brandolini, 2006), contrary to American popcorn, which became a prominent commercial crop. Despite their germplasm diversity, Italian popcorns were never considered in the genetic improvement programs of the 20th century and thus do not appear in major literature sources (Zapparoli, 1930; Brandolini and Brandolini, 2006, 2009).

Currently, very fragmented knowledge is available about Italian popcorn germplasm; Brandolini and Brandolini (2006) reported a sampling, though incomplete, conducted by Aureliano Brandolini on popcorn in Northern Italy between the 1970s and 1990s. This sampling recovered 47 accessions of popcorn maize, which were divided into three distinct categories based on kernel shape: “Perla”, “Orizoide” and “Cuspidato Bianco” (respectively “pearl-type”, “rice-type”, and “white-cusp-type”); unfortunately these materials have been lost (Andrea Brandolini, personal communication), and a new Italian collection is currently hosts at the Germplasm Bank of Pavia University (infrastructure part of Aegis European Integrated System) for long term conservation. Additionally, two recent studies have compiled comprehensive and updated inventories of Italian traditional popcorns, their uses, and their history to characterize and enhance these materials. In 2019, Ardenghi and colleagues, from Pavia University, conducted a survey that combined the systematic collection of oral testimonies with the physical sampling of popcorn seeds (Rossi et al., 2019). This research yielded two main outcomes: firstly, they gathered 26 oral testimonies indicating the presence of popcorn landraces in nine different regions, from Northern to Southern Italy, proving the widespread distribution of local popcorns in the past, passed down through generations since at least the late 19th century. Secondly, they acquired seeds of 15 accessions from seven Italian regions (Piemonte, Lombardia, Veneto, Trentino-Alto Adige, Emilia-Romagna, Basilicata and Campania). These samples can be referred to the two main types of popcorn (“rice-type” with pointed kernels, and “pearl-type” with rounded kernels) mentioned by Brandolini and Brandolini (2006). In addition, Stagnati et al. (2021) described a popcorn variety from Emilia-Romagna, while Canella et al. (2022) identified and briefly characterized eight popcorn landraces distributed across four regions (Emilia-Romagna, Lombardia, Trentino-Alto Adige, and Veneto). Among these eight landraces, six are cultivated on a horticultural scale by amateurs for personal consumption, while the remaining two are cultivated and also commercialized on a small scale in mountain agriculture. These studies focusing on the Italian local popcorn germplasm demonstrate that, like all landraces, these varieties are deeply connected to the customs and traditions of the regions where they are cultivated, possessing significant historical as well as genetic value (Lezzi et al., 2023). Additionally, since popcorn landraces vary from region to region based on ecological conditions, ability of the cultivar adaptation, and consumers’ preferences (Ziegler et al., 1984), they presumably harbor enormous genetic diversity that offers multiple opportunities for genetic enhancement (Lezzi et al., 2023; Gopinath et al., 2023).

In Europe, the popcorns have been poorly investigated. Morphologic and genetic characterization of 626 European maize landraces included only 14 landraces genetically assigned to popcorns (Balconi et al., 2024) while Gouesnard et al. (2017) analyzed 1191 European Flint lines including 33 popcorn from those only 6 bred in Europe, specifically Romania and Bulgaria. Of these landraces or inbred none are from Italy, thus confirming the scarce knowledge on this maize type even if some Italian landrace have genetic contribution form popcorn (Balconi et al., 2024).

Considering that the conservation status of all these local Italian popcorn varieties is at high risk of extinction and there is no available knowledge on Italian popcorns, the present study collected and characterized ten different popcorn landraces from Northern Italy, some of them still cultivated on farm.

## 2 Materials and methods

### 2.1 Germplasm and field management

Ten traditional maize landraces of popcorn from Northern Italy were retrieved from the germplasm collections of the Plant Germplasm Bank of the Department of Earth and Environmental Sciences at Università degli Studi di Pavia (Pavia, Italy) and of the Department of Sustainable Crop Production of Università Cattolica del Sacro Cuore (Piacenza, Italy). Detailed information on the maize germplasm used in this study are provided in Supplementary **Supplementary Table S1**.

Maize accessions were reproduced by manual random intermating, avoiding self-pollination. Seed stocks are stored at Plant Germplasm Bank of the Department of Earth and Environmental Sciences at Università degli Studi di Pavia (Pavia, Italy) as well as at the the Department of Sustainable Crop Production at Università Cattolica del Sacro Cuore (Piacenza, Italy).

Field trials followed a completely randomized design with three replications originated by the *blockdesign* package (Edmondson, 2020) of the R software (R Core Team, 2023) and were conducted at CREI-CERZOO (45°0.303960′ N, 9°42.252360′ E, San Bonico, Piacenza, Italy). The same randomized field trial was sown on April 23^rd^ and April 20^th^ in 2021 and 2022, respectively, and managed according to appropriate agricultural practices for maize nursery cultivation. Herbicides were not applied and weed management was done manually as required.

Each plot consisted of 4 rows 6 m long, spaced 80 cm apart from each row, and 1 m aisle on the hedge; for each row, 25 seeds were planted. For each plot, the two central rows were used in order to assess agronomic traits relying on the indications of the UPOV-CPVO TP/2/3 protocol as follows: tasseling (50% of plants shedding pollen), silking (50% of plants with visible silks), and physiological maturity (presence of the black layer). Plant height (tassel included) and ear eight (primary ear) were measured on five plants; lodging was evaluated three weeks after flowering. To estimate yield potential of popcorn landraces, ears were hand harvested and manually shelled; grain moisture was calculated to adjust yield potential at 15.5% of moisture to a final plant density of 50 thousand plants/hectare. Moreover, the thousand kernel weight (TKW) was measured, the percentage of popping kernels was evaluated by popping 200 seeds using a domestic popcorn machine, and the volume increase from dry seed (SV) to popped flake (PV) volumes were measured using a graduated glass cylinder to calculate the volume increase according to ((PV-SV)/SV)*100.

To evaluate the susceptibility to *Fusarium verticillioides* artificial inoculation was performed according to the pin-bar inoculation method at 15 days after pollination (DAP) following the procedure described by Stagnati et al. (2024) and Guche et al. (2022). The inoculum was produced as described in Lanubile et al. (2013, 2021) to a final concentration of 1 × 10^6^ conidia/mL. Inoculated ears were hand harvested at maturity and air dried in a barn.

Fusarium ear rot (FER) severity was visually evaluated, assessing the percentage of the rotted surface of the ear (Stagnati et al., 2020a, 2024; Guche et al., 2022). In addition to inoculated ears, FER was evaluated also on naturally infected ears.

### 2.2 Plant material and DNA extraction

Leaf samples were collected in 2022, considering 28 individuals per landrace, except for Popcorn Caravaggio for which the individuals were 30, resulting in a total of 282 samples. Genomic DNA was extracted with GenElute™ Plant Genomic DNA Miniprep Kit (Merck Life Science s.r.l., Darmstadt, Germany) following manufacturer instructions with minor corrections as reported in Stagnati et al. (2021). The extracted DNA was then evaluated for quality and quantified using NanoPhotometer N80 (Implen GmbH München, Germany) and visualized on 1% agarose gel electrophoresis stained with Eurosafe nucleic acid stain (EuroClone S.p.A., Pero (MI), Italy).

### 2.3 Sequencing and bioinformatic analysis

Genotyping, raw reads processing and variant calling were performed at CD Genomics – The Genomics Services Company (SUITE 111, 17 Ramsey Road, Shirley, NY 11967, USA) with double digest Restriction Associated DNA sequencing approach (ddRAD) (Peterson et al., 2012) using ApeKI as restriction enzyme (Elshire et al., 2011). Pair-end sequencing was performed on Illumina^®^ HiSeq PE150 platform (Illumina, California, USA). The original sequencing data acquired by high-throughput sequencing platforms were transformed to sequence reads by base calling with the CASAVA software. Raw data obtained from sequencing were filtered for adapter contamination and reads with more that 10% of uncertain nucleotides and base quality less than 5 reads (Q ≤5) were discarded.

The resulting clean reads were mapped against the Maize B73 RefGen_v5 Genome (reference genome: GCA_902167145, Harper et al., 2016) using BWA software (Li and Durbin, 2009). Duplicates in the alignment results were removed by SAMTOOLS, and the mapping rate and coverage was counted according to the alignment results (Li et al., 2009).

Finally, single nucleotide polymorphisms (SNPs) were detected and analyzed using GATK, resulting in a set of 5,826,704 variant SNPs, which were used for downstream analyses. SNPs distribution on maize genome was visualized using CMplot package on R-studio (Yin et al., 2021; R Core Team, 2023); SNPs were further filtered out removing those variants with minor allele frequency (MAF) lower than 0.05 (Caproni et al., 2023) and with missing call rates higher than 0.20 (Abebe et al., 2015; Stagnati et al., 2019, 2020b). In addition, a linkage disequilibrium (LD) pruning step was performed with the command –indep 150 5 0.5 of the PLINK software, as recommended in the PLINK manual (Purcell et al., 2007) and in a previous study (Calus and Vandenplas, 2018).

### 2.4 Population genetics and statistical analysis

Genetic structure and diversity of maize varieties under study were elucidated through principal component analysis (PCA) (Greenacre et al., 2022; Lezzi et al., 2023) using PLINK v.1.9 (Chang et al., 2015) to principal components calculation. R software v. 4.1.3. was used for visualizing the resulting plot. Population structure was investigated using the ADMIXTURE v.1.3.0 package (Alexander et al., 2020), with the number of clusters (K) ranging from 2 to 15. To assess the quality of the clustering and infer the most likely K-value, the cross-validation error for each K-value was estimated. The results of the ADMIXTURE analysis were visualized using the *Pophelper* R package (Francis, 2017).

To depict the phylogenetic relationships among individuals, a phylogenetic tree was constructed with *ape* R package (Paradis and Schliep, 2019) through the UPGMA algorithm with 100 bootstrap replicates to assess branch support.

Observed and expected heterozygosity, inbreeding coefficient (F), and population pairwise fixation index (F_ST_, Weir and Cockerham, 1984) were computed with VCFtools (Danecek et al., 2011) using the “–het” and “–weir-fst-pop” functions, respectively (Dominguez et al., 2024). Graphs were subsequently plotted with *ggplot2* and *heatmap.2* packages in R (Wickham, 2014; R Core Team, 2023; Warnes et al., 2024).

Field data visualization and analysis was performed in R software v. 4.3.0. Correlation among traits were investigated by the *corrplot* package (Wei and Simko, 2021), principal component analysis was visualized with the *factoextra* package (Kassambara and Mundt, 2020), while ANOVA and Tukey test were conducted using *car* and *agricolae* packages (Fox and Weisberg, 2019; de Mendiburu, 2023). Before ANOVA, data were transformed according to the Box-Cox transformation function available in the *Mass* package (Venables and Ripley, 2002). A +1 constant was added to account for zeros in lodging percentage and FER incidence without inoculation (N_FER).

## 3 Results

### 3.1 Historical and morphological characterization of germplasm

Morphological characters were measured and used to describe accessions; plant descriptors are reported in Supplementary **Supplementary Tables S2–S11**. Historical information was retrieved by direct interviews with germplasm donors and their families.

The landrace Popcorn di Torre d’Isola, which includes three different accessions named Torre d’Isola Bianco, Torre d’Isola Blu and Torre d’Isola Rosso, was sampled in 2018 in Torre d’Isola village (Pavia, Italy). This landrace can be referred to “pearl type” popping maize according to Brandolini and Brandolini (2006), characterized by its small, rounded kernels. The three strains are differentiated by kernel color, which is white, blue or red. Cultivation of progenies separated by seed color revealed some differences among the three types. Morphologically, the plants reach flowering between 80–90 days after sowing. Tillers are almost absent in white and red derived progenies, while the blue type tends to tiller to a limited extent. The ears are relatively short: 13 cm in the red-seeded type, 11 cm in the white-seeded type, and 8 cm in the blue-seeded type. The number of rows per ear varies between 14 and 18. The cob is reddish in all three kernel color progenies. The popped kernels produce white, butterfly-shaped flakes. Specifically, Torre d’Isola Bianco (**Supplementary Table S2**) has an average plant height of 210 cm and a first ear insertion height of 128 cm, resulting in an ear-plant insertion ratio of 61%. The male inflorescence has a strong glumes anthocyanin pigmentation, but absent pigmentation at the base of the glumes, and moderate pigmentation on the anthers. Female inflorescences display moderate or strong silks’ pigmentation. Torre d’Isola Blu (**Supplementary Table S3**), instead, has an average plant height of 183 cm and a first ear insertion height of 90 cm, resulting in an ear-plant insertion ratio of 54%. Tassels display absent anthocyanin pigmentation at ring at the base of the glumes and weak pigmentation of glumes and anthers. Female inflorescences do not display silks’ pigmentation. Finally, Torre d’Isola Rosso (**Supplementary Table S4**), has an average plant height of about 200 cm and a first ear insertion height of 117 cm, resulting in an ear-plant insertion ratio of 58%. Tassels display absent to weak anthocyanin pigmentation on the ring at the base of the glumes, on the glumes and the anthers. Female inflorescences display a weak silks’ pigmentation. Brandolini and Brandolini (2006) reported that in the areas of Novara (near Piedmont) and Vigevano (PV), pearl-type popping maize with red or white kernels was cultivated, showing a high affinity with the red and white lines of the Torre d’Isola maize. This variety was traditionally cultivated in Cascina Scaldasole since 1938, but now stopped since the only one old farmer is now dead.

Concerning the landrace named Perla di Quarona, seeds were acquired through exchanges with amateur growers of local varieties from Val Brembana and Val Seriana in Bergamo Province (Lombardy). The name refers to a landrace described by Brandolini and Brandolini (2006) which is morphologically comparable to the present accession (**Supplementary Table S5**), also if Quarona is located in Vercelli province (Piedmont). This landrace is a type of pearl popping maize with small, rounded kernels. The plants grow to a height of 180 cm, with the first ear positioned high on the stalk at 130 cm, resulting in an ear-plant insertion ratio of 74%. It is a late variety flowering approximately 100 days after planting. The plants produce tillers that fully develop, each stalk bearing 2–3 ears, 9 cm long with 12–14 rows of small blue kernels. The cob is white, and the popped kernels are white of butterfly or intermediate shape. The male inflorescence shows absent anthocyanin pigmentation on the ring at the base of the glumes and on anthers, and a weak pigmentation on glumes. Female inflorescences do not display silks’ pigmentation.

In San Martino Siccomario (Pavia, Italy) a landrace was sampled in 2018. There are two forms of this pearl-type popcorn: a typical form with only dark-colored ears (Nero di San Martino Siccomario) and a variegated form with some light-colored kernels (Variegato di San Martino Siccomario). Both types are maintained to preserve the dual coloration on the ear and are morphologically very similar. The plants are often tillered, about 200 cm tall, with the first ear positioned at 135 cm on the stalk, and they have a late flowering period of approximately 100 days after planting. The ears are generally numerous (up to 6-7), 8 cm long with 12–14 rows of blue kernels in the Nero, while the Variegated type has 14–16 rows of kernels segregating white/blue colors on a white cob in both variants. Popped kernels are of butterfly type while in mushroom shape appears occasionally in the Nero variant. Specifically, Nero di San Martino Siccomario (**Supplementary Table S6**) has an average plant height of 250 cm and a first ear insertion height of 137 cm, resulting in an ear-plant insertion ratio of 67%. The male inflorescence shows absent anthocyanin pigmentation on the ring at the base of the glumes and on anthers, and an absent to weak pigmentation on glumes. Similarly to the tassel, also silks display an absent pigmentation. Variegato di San Martino Siccomario (**Supplementary Table S7**), instead, has an average plant height of 200 cm and a first ear insertion height of 133 cm, resulting in an ear-plant insertion ratio of 64%. As before, male inflorescence shows absent anthocyanin pigmentation on the ring at the base of the glumes and on anthers, and an absent to weak pigmentation on glumes. Female inflorescences do not display silks’ pigmentation. Small-sized blue pearl popping maize (kernel and ear) was probably widespread at least since the post-World War II era in the area between the Po and Ticino rivers, enclosed by the railway connecting the two rivers. Currently, it is documented to be cultivated domestically in San Martino Siccomario (PV).

The Popcorn accession Suzzara was recovered in Vespolate (Province of Novara, Italy) in 2019, also if its origin is presumably from Suzzara (Mantua, Lombardy), here transferred from the only family cultivating this accession. This is a popcorn variety with yellow “rice-shaped” kernels (Brandolini and Brandolini, 2006). The plants are about 160 cm tall, with the ear positioned at mid-height (78 cm), resulting in an ear-plant insertion ratio of 48%. The variety flowers at 85 days after planting and matures in 130 days. The ears are 12 cm long and have 10–14 rows of yellow kernels on a white cob. After popping, flakes are of white-cream color and of butterfly. The male inflorescence shows moderate anthocyanin pigmentation on the ring at the base of the glumes, and absent pigmentation on glumes and on anthers. Female inflorescences do not display silks’ pigmentation (**Supplementary Table S8**). This maize bears a slight resemblance to a late white “rice-shaped” popcorn from Cremona described in Brandolini and Brandolini (2006). It is presumably the oldest known Lombardy popcorn variety. Currently, it is cultivated in Terdobbiate (Novara) and trace back to cultivation in Suzzara (Mantova) in the early 1900s.

Popcorn di Caravaggio was collected in 2020 in Caravaggio (Bergamo, Italy). This variety flowers earlier than others, around 80 days after sowing. The plants are about 160 cm tall, with the first ear positioned 76 cm above the ground, resulting in an ear-plant insertion ratio of 47%. The ears are 14 cm long, with 12–18 rows of rounded, yellow-orange kernels on a white cob. The popped kernels are generally butterfly-shaped, sometimes intermediate and have an appealing white-cream color. The male inflorescence shows absent to weak anthocyanin pigmentation on the ring at the base of the glumes, and absent pigmentation on glumes and on anthers. Female inflorescences do not display silks’ pigmentation (**Supplementary Table S9**).

Popcorn Spose del Primiero was retrieved in 2019 in Imèr (Province of Trento, Italy). The name “Spose” (brides) may refer both to the color of the seed and the flakes while “Primiero” refers to the valley of Cismon river, defiled by Pale di San Martino and Lagorai mountains in the Dolomites. This variety flowers earlier than others, around 65–70 days after sowing. The plants are 175 cm tall, with the first ear positioned 70 cm above the ground, resulting in an ear-plant insertion ratio of 43%. The ears are 11.8 cm long, with 14.18 rows of rice-shaped, white-yellowish kernels on a white cob. The popped kernels are generally butterfly-shaped and of white color. The male inflorescence shows absent to weak anthocyanin pigmentation on the ring at the base of the glumes, and absent pigmentation on glumes and on anthers. Female inflorescences do not display silks’ pigmentation (**Supplementary Table S10**).

The last popcorn landraces is Muneghe Nere that was collected in 2018 in Seren del Grappa (Belluno, Italy). The name, literally “Black Nuns” is of unclear etymology. The use of “muneghe” and similar words is common in Veneto region, its association with “black” may underline the contrast of the dark seed color and the white flakes after popping. The variety flowers around 100 days after planting. The plants are about 150 cm tall, with the first ear positioned 90 cm above the ground, resulting in an ear-plant insertion ratio higher than 67%. The ears are 9.6 cm long, with 12–14 rows of rounded, black kernels on a white cob. The pop flakes are white and butterfly-shaped. The male inflorescence shows absent anthocyanin pigmentation on the ring at the base of the glumes and on the anthers, while weak pigmentation can be observed on glumes. Female inflorescences do not display silks’ pigmentation (**Supplementary Table S11**).

### 3.2 Agronomic characterization

**Figure 1:**
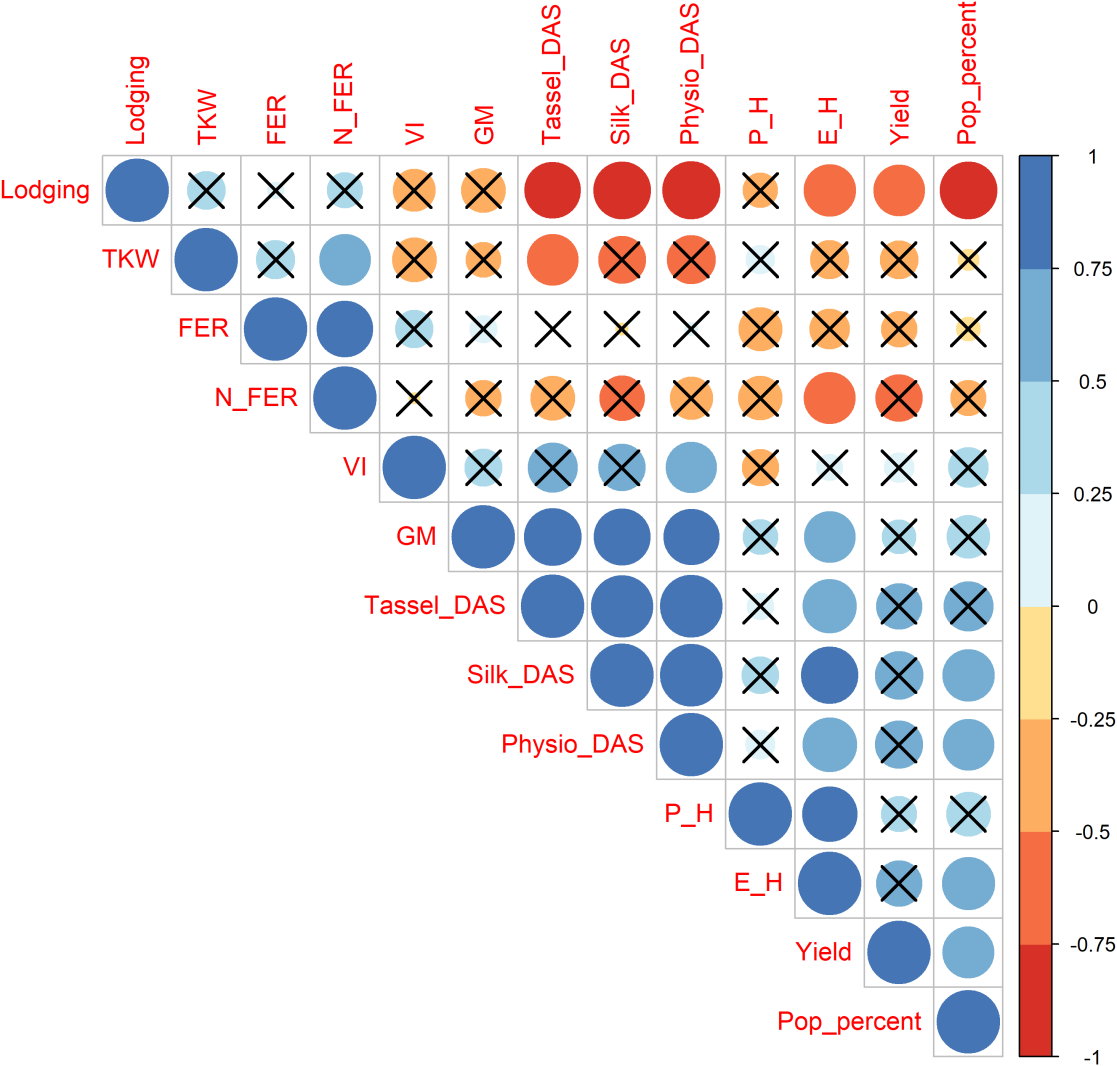
Correlation among phenotypic traits measured over two growing seasons in ten maize landraces of Italian popcorn.

Agronomic characterization conducted over two consecutive seasons revealed significant differences among genotypes and years (**Table 1**). Strong positive correlations (**Figure 1**) exist between flowering and maturity time, which are both dependent by genotype and environment. The earliest landrace was Spose del Primiero, while the latest ones Muneghe Nere, Perla di Quarona, and both Nero and Variegato di San Martino Siccomario (**Table 1**). Lodging is a key factor that drives the agronomic success of maize landrace: strong and negative correlation is detected among flowering time and lodging susceptibility. Significant differences exist among genotypes (p-value 9.86*10^-5^) with the most susceptible being Spose del Primiero and Caravaggio, while for the other landraces lodging is almost negligible (**Table 1**). Plant and ear insertion height are only influenced by the genotype (p-value 4.02*10–13 and 5.17*10-12, respectively); interestingly, ear height was negatively correlated to lodging (**Figure 1**). Yield is limited but differences are relevant among varieties (9.96*10^-5^) ranging from 0.24 t/ha for Spose del Primiero to 4.32 t/ha for Torre d’Isola Rosso with lodging affecting severely the yield (**Figure 1**). Popcorns stay in the field longer compared to standard corn, because they are harvested at 16% moisture (Ziegler, 2000) and this trait is affected both by genotypes and year (p-value 1.25*10–^5^ and < 2*10^-16^, respectively) and well as by genotype per environment interaction (**Table 1**) being higher for long cycle landraces. To better understand the agronomic potential of popcorn landraces, and to investigate the potential as source of disease resistance we investigated the susceptibility of these landraces to Fusarium Ear Rot both under natural and artificial inoculation. These two traits are highly correlated (**Figure 1**), influenced by genotype (p-value 0.00684 and 0.00219, respectively) and uncorrelated to flowering; the most susceptible landrace being Suzzara with a 57% of disease infection while the most promising landraces are the Torre d’Isola strains: Bianco (7.75%), Rosso (14.02%) and Blu (18.3%).

**Table 1:** Mean values, two-way ANOVA and Fisher’s l.s.d. test of phenotypic data collected from 10 popcorn landraces evaluated, considering both growing seasons (2021 and 2022).

Variety	Tassel DAS	Silk DAS	Physiologic maturity DAS	Lodging	GM	Yield	FER	N_FER	P_H	E_H	Pop %	VI	TKW
Muneghe Nere	98.83^a^	92.50^ab^	136.33 ^ab^	0.00^c^	12.59^abc^	2.61^ab^	32.40^ab^	6.77^ab^	148.97^d^	99.30^bc^	80.67^bc^	1707.35^ab^	65.38^d^
Nero SMS	97.83^a^	99.83^a^	140.16^a^	0.00^c^	12.84^ab^	2.50^abc^	35.63^ab^	2.52^b^	205.30^a^	137.40^a^	83.30^abc^	1656.44^ab^	86.38^bc^
Perla Quarona	98.17^a^	99.00^a^	138.00^a^	0.00^c^	12.41^abcd^	3.15^ab^	24.79^ab^	3.20^b^	179.43^c^	131.43^a^	84.50^ab^	1861.62^a^	65.23^d^
Caravaggio	75.83^d^	76.67^de^	121.66^cd^	9.67^ab^	11.67^d^	2.49^bc^	25.32^ab^	3.70^ab^	162.63^cd^	76.50^c^	69.83^c^	1571.79^b^	84.93^c^
Spose	65.33^e^	67.83^e^	112.75^d^	12.00^a^	11.77^bcd^	0.24^c^	37.00^ab^	24.86^ab^	175.27^cd^	75.67^c^	57.83^c^	507.83^f^	111.89^a^
Torre Isola Bianco	83.83^b^	86.00^bc^	127.16^c^	0.83^bc^	11.93^bcd^	2.24^bc^	7.75^b^	1.31^b^	211.27^a^	128.40^a^	94.00^a^	1204.86^cd^	95.23^bc^
Torre Isola Blu	84.00^bc^	88.17^bc^	129.00^bc^	0.50^bc^	11.73^cd^	2.96^ab^	18.30^b^	3.87^b^	183.47^bc^	99.87^bc^	74.42^bc^	922.99^de^	86.03^bc^
Torre Isola Rosso	79.83^cd^	82.83^cd^	125.83^c^	1.83^ab^	11.78^cd^	4.32^a^	14.02^b^	1.47^b^	201.57^ab^	117.27^ab^	86.42^ab^	786.15^ef^	94.29^bc^
Variegato SMS	98.67^a^	100.33^a^	137.66^a^	0.00^c^	13.38^a^	3.63^ab^	33.78^ab^	3.80^b^	206.93^a^	133.40^a^	93.58^a^	1256.13^c^	97.13^b^
Suzzara	80.00^bcd^	82.50^cd^	127.50^c^	0.67^bc^	11.78^cd^	2.43^bc^	57.13^ab^	34.17^a^	161.50^cd^	78.60^c^	87.00^ab^	1780.48^ab^	114.18^a^
lambda	2.30	2.10	4.7	-1.90	-3.10	1.00	#	-0.30	1.80	1.70	2.80	1.10	1.00
G	< 2*10^-16^	< 2*10^-16^	< 2*10^-12^	9. 10^-5^	1.25*10^-05^	9.96*10^-05^	0.00684	0.00219	4.02*10^-13^	5.17*10^-12^	1.38*10^-07^	< 2e-16	< 2e-16
Y	5.86*10^-6^	5.86*10^-6^	0.15^6^	0.18	< 2*10^-16^	0.379	0.46227	0.10643	0.276	0.174	< 2*10^-16^	2.44*10^-5^	0.0107
G*Y	0.0129	0.18	0.0029	0.263	4.7910^-2^	0.09	0.70035	0.15109	0.81	0.978	0.01	6.26*10^-10^	0.3404

DAS, Days After Sowing; GM, Grain moisture; FER and N_FER, Fusarium Ear Rot severity after artificial or natural (N) inoculation; P_H and E_H, plant (P) and ear € height; VI, volume increase after popping; TKW, thousand kernel weight.Superscript letters and numbers are related to statistical analysis (see section 2.4).The meaning of the symbol “*” in **Table 1** is “by”.

**Figure 2:**
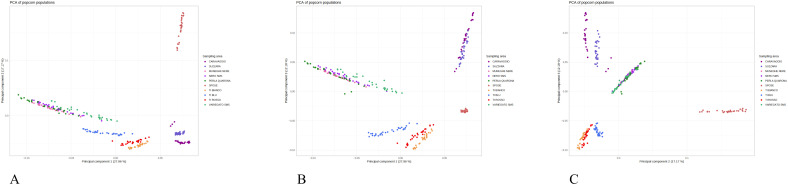
Principal component analysis based on genetic markers of popcorn landraces from Italy. **(A)** Coordinate 1 vs. Coordinate 2 of the 279 samples characterized by the 875,829 SNPs set; **(B)** Coordinate 2 vs. Coordinate 3; **(C)** Coordinate 3 vs. Coordinate 1.

In addition, popcorn making was evaluated by measuring TKW, popping percent and volume increase after seed popping. These traits are uncorrelated and associated to different popcorn landraces (**Figures 1**, **2**). Significant differences exist among landraces and years with significant G*Y interaction for volume increase (**Table 1**). The worst landrace for popping percent and volume increase is Spose del Primiero, which has other agronomic defects, while the best genotypes are Torre d’Isola Bianco and Variegato di San Martino Siccomario for popping percent, and Perla di Quarona for volume increase. The situation is opposite for TKW with Spose del Primiero being the best landrace.

Even though the number of landraces investigated is limited, the phenotypic diversity is relevant on the most important traits and each landrace is distinguished from the other because of features (**Supplementary Figure S1**). Phenotypic clusterization explains 54.7% and 19% of variation for the first two principal components, respectively. It is possible to distinguish a cluster formed by both San Martino Siccomario and Perla di Quarona and another one where Torre d’Isola Bianco and Rosso are more closed. Such clusterization may suggest genetic relatedness among those landraces rather than the others that appear as single entities.

### 3.3 Sequencing and bioinformatic analysis

The GBS of the 282 maize samples yielded 5,826,704 variants well distributed along the entire genome (**Supplementary Figure S2a**). Three samples were removed from the dataset due to a lack of information. Maize data set was then filtered for a minor allele frequency (MAF) of 0.05, obtaining 875,829 SNPs, which were further filtered for linkage disequilibrium with an r² threshold of 0.5 producing a final number of 313,342 variants that have been visualized on the maize genome (**Supplementary Figure S2b**).

From the phylogenetic tree, it is evident that the most genetically distinct population is Spose del Primiero (**Supplementary Figure S3**). Following the divergence of the Spose del Primiero popcorn, two further branches form. The first branch includes two yellow-seeded landraces of the lowlands: Popcorn di Suzzara and Popcorn di Caravaggio. The second branch splits further into two sub-branches. On one side are the three types of Popcorn di Torre d’Isola, with the blue-seeded variant being more distant from the red and white-seeded variants. The popcorn landraces of San Martino Siccomario, Muneghe Nere, and Perla di Quarona are on the other side. The two San Martino Siccomario types are distinctly separated from each other and from other maize types, despite the recent divergence between the Nero and Variegato landraces. In contrast, Perla di Quarona and Muneghe Nere, although originating from geographically distant regions, do not show a clear separation as it might have been expected.

The PCA based on the genetic markers, explains in its first and second components 27.96% and 17.17% of the total variance, respectively, while the third one accounts for 12.18% (**Figure 2**). The PCA based on genetic markers results are largely consistent with those of the phylogenetic tree: Spose del Primiero landrace is the most distinct from the group, popcorn di Caravaggio and Popcorn di Suzzara are closely clustered, and popcorn di Torre d’Isola are grouped but sufficiently distinct, with the blue-seeded variant more distant from the red and white-seeded variants and close to the prolific types. These prolific types, represented by Muneghe Nere, Perla di Quarona, and San Martino Siccomario (both the Nero and Variegato types), appear as a single group. Even when the orientation of the PCA is changed, the group of prolific popcorn varieties remains intact.

The population structure analysis (**Supplementary Figure S4**) identified eight ancestral populations (CV error = 0.61) that contributed to the present collection with findings consistent to the PCA and phylogenetic tree.

The germplasm collection showed a very high inbreeding coefficient ranging from 0.91 (Perla di Quarona) to 0.95 (Torre d’Isola Bianco); the same two landraces are those with the lowest (0.013) and highest (0.22) observed heterozygosity (**Supplementary Figures S5**, **S6**). The comparison between expected and observed heterozygosity revealed a significant (p < 0.005) lack of heterozygosity for all the ten popcorn landraces. Fixation index (F_ST_) provides a measure of genetic differentiation; F_ST_ value across the collection, visualized by heatmap (**Supplementary Figure S7**), showed a mean of 0.166 ranging from 0.01 (Muneghe Nere vs. Perla di Quarona) to 0.25 (Muneghe Nere vs. Spose), very small values were found also for Torre d’Isola Rosso vs. Torre d’Isola Bianco and Torre d’Isola Rosso vs. Torre d’Isola Blu (0.04 and 0.08).

## 4 Discussion

Popcorn is very well-known and loved for its kernels’ main agronomic trait: the expansion when heated. This distinctive characteristic made this maize type extremely important on the commercial point of view, even if the intensive cultivation spread mostly in the United States (Kaur et al., 2021).

In Italy, popcorn local varieties remained confined to few family gardens as comfort food, never gaining the role of commercial crop, nevertheless its history is very old in the country (Zapparoli, 1930; Brandolini and Brandolini, 2006).

At present time, very little literature could be found specifically on Italian popcorns (Brandolini and Brandolini, 2006; Ardenghi et al., 2019; Rossi et al., 2019; Stagnati et al., 2021; Canella et al., 2022), aiming to fill this gap in the present research was conducted an historic, morphologic and genetic characterization of ten popcorn landraces originating from the Northern part of Italy.

Starting with the agronomic traits, the morphologic characterization of the landraces under study revealed significant differences among genotypes. At an inter-population level, the ten landraces appear to be distinct, and at intra-population level, they seem to be uniform.

Our characterization revealed two interesting traits, the first one is that, according with previous studies such as the one from Balconi et al., 2024, popcorns have longer cycles compared to other Italian landraces. Moreover, two popcorn landraces (Spose del Primiero and Caravaggio) have been identified as susceptible to lodging.

Agronomic performances of popcorn varieties are lower compared to other corn types (Babu et al., 2006), moreover ear are inserted higher on the plant and in many cases the popcorn stalk is weaker than other maize types (Ziegler et al., 1984; Babu et al., 2006; Balconi et al., 2024; Stagnati et al., 2024). Since these features may contribute to increase lodging susceptibility, crosses with dent corn are often practiced in breeding programs to improve agronomic value of popcorn, even if several backcrosses and selection are then required to recover typical traits to popcorn (Babu et al., 2006). In the present collection, there are some promising landraces with good lodging resistance (particularly Muneghe Nere, Perla di Quarona, Nero and Variegato di San Martino Siccomario) that can be of interest for future improvements.

Another very interesting trait to investigate is the susceptibility of these landraces to Fusarium Ear Rot both under natural and artificial inoculation, which is important because popcorn is intended for human consumption. de Almeida et al. (2023) conducted a three-step evaluation of South American popcorn lines to identify resistance sources to use to breed superior hybrids selecting under natural and artificial inoculation while do Nascimento Ferreira Kurosawa et al. (2017) evaluated 37 South-American accessions to identify promising sources for resistance. It is believed that hard endosperm is more favorable for ear rot resistance which still remain a complex trait to elucidate and improve (Santiago et al., 2015) The correlation among natural and artificial inoculation has been previously reported (Stagnati et al., 2020a, b), while De Almeida Silva et al. (2020) reports that selecting for yield and popping traits reduces also the susceptibility to FER. In the present collection, FER resistance seemed to be uncorrelated to other traits suggesting the possible use of the most tolerant landraces to extract superior inbred lines without detrimental effects on other traits.

Traits that are relevant for popcorn marketability are TKW, popping percent and volume increase after seed popping, which were also evaluated in the present study, and significant differences among landraces and cultivation years were found. The worst landrace for popping percent and volume increase is Spose del Primiero, which has moreover other agronomic defects. The best genotypes are Torre d’Isola Bianco and Variegato di San Martino Siccomario, which have also good potential for agronomic traits. When analyzed, the increase of the volume after popping resulted to be the best in the landrace be Perla di Quarona. Opposite results can be seen in TKW measures with Spose del Primiero being the best landrace. Landraces are adapted to the peculiar condition of their area of origin and may behave differently in other areas. The presence of G*E interaction for these traits may suggest that in their typical area they can have superior performance. Defying a good quality standard in popcorn is challenging because of the many end-used groups in popcorn making. A popcorn consumer’s definition of high quality is a product that has a good appearance, flavor and texture, freedom from hulls and un-popped kernels (Ziegler, 2000). This definition applies well to landrace valorization, where the market is direct from grower to consumer. Final consumers prefer popcorn with a butterfly shape because they are associated with tenderness compared to the mushroom shape, which is appreciated by industry. To this, it appears that all the landraces have been unintentionally selected to reach this flake shape. On the other hand, from breeder’s perspective, the most important trait to evaluate is popping expansion, which in the present collection revealed significant differences among varieties.

Finally, it can also be said that historical and morphological characterization of the landraces under study correspond to the genetic one. Spose del Primiero popcorn appears to have no relationship with other accessions, consistent with the findings from the phylogenetic tree, PCA, and F_ST_ pairwise comparison. This is the earliest-maturing landrace among those studied, with white, rice-shaped kernels, originating from Imèr, in the province of Trento. The remote origin may have contributed to the genetic distance and isolation to other accessions. Population structure analysis further allowed the separation of the prolific group: Muneghe Nere and Perla di Quarona popcorn varieties seem to share the same origin, which aligns with the PCA and phylogenetic tree results, as well as to the F_ST_ differentiation. San Martino Siccomario landraces, clustered with Muneghe Nere and Perla di Quarona in PCA and phylogenetic tree analysis, whereas are separated from the other two populations in population structure analysis. F_ST_ pairwise comparisons support population structure analysis’ results as it can be seen that landrace Nero di San Martino Siccomario shares more similarities with Muneghe Nere and Perla di Quarona, than Variegato di San Martino Siccomario does with the two landraces mentioned above. The typical San Martino Siccomario popcorn differs slightly from the variegated form, which shows a mixed origin with significant contributions from two different populations (red and orange). Similarly, the three popcorn Torre d’Isola, although cultivated together, exhibit genetic differences: Torre d’Isola Bianco and Torre d’Isola Blu popcorn both originate from two populations, while the Torre d’Isola Rosso popcorn derives from three different ancestral populations. This finding again is consistent with the F_ST_ differentiation results, which showed high genetic similarity between Torre d’Isola Rosso with both Torre d’Isola Bianco and Torre d’Isola Blu, while the latter two landraces seems to be more dissimilar one from the other. Finally, despite being grouped together in the PCA and phylogenetic tree, with a low F_ST_ value, Suzzara and Caravaggio landraces are separated by the population structure analysis, although they share a minimal contribution from the orange ancestral population.

Maize is an outbreeding crop and it is expected that single individuals within the same landrace are highly heterozygous (Hölker et al., 2019; Balconi et al., 2024). Moreover, observed heterozygosity lower than 0.1 suggest that local populations have not been well maintained (Balconi et al., 2024), as a possible consequence of the agricultural practices often linked often with these materials, i.e. (i) cultivation confined in very small parcels reduced to few plants enough for domestic usage and not as staples or markets; (ii) farmer habit to save the seed for the subsequent generation from single or few ears thus applying a very strong unwanted selection. These two aspects together can explain the high homozygosity because having only a very low genetic variability in the garden, coupled with saving seed from single ears for the subsequent season, can result, year after year, in an increment of inbreeding and a reduction of heterozygosity.

It cannot be excluded that, for many generations, the cultivation of a very small number of individuals and the habits of saving seeds from one-few ears Canella et al. (2022) reported that Italian popcorn landraces are mainly grown at horticultural scale for personal consumption. The high level of homozygosity could also derive from strong bottleneck during their initial collection (Balconi et al., 2024) and both motivations find confirmation with the high morphological uniformity within each popcorn landrace. Selection impact on genetic diversity, it has been reported that recurrent selection reduced expected heterozygosity to an extent of around 40% after 12 cycles in Iowa Stiff Stalk Synthetic and Iowa Corn Borer Synthetic maize population and similar results have been evidenced also in tropical maize breeding populations (Alves et al., 2018).

To conclude, the present work examined a popcorn landrace collection *ex situ* preserved from northern Italy, mainly from Lombardy. Morphological characterization allowed an accurate landrace description and subsequent agronomic comparison with evaluation of performances of these materials in modern agricultural context. Phenotypic variability exists among genotypes and significant differences have been detected for all agronomic traits. This suggests the possible presence of adaptation to conditions typical of the landrace area of origin. Moreover, we carried out a genetic characterization, which highlighted relevant variability among landraces. Clusterization and population structure analysis revealed interesting patterning consistent to the available historical information and morphological observations. This collection evidenced high inbreeding levels of the materials, which seems to be more similar to inbred lines than to typical landraces. Moreover, the high similarity between Muneghe Nere and Perla di Quarona landraces suggests that they could derive from the same genetic background as a consequence of seed exchange in the past. This situation deserves more investigation because the origin of Perla di Quarona refers to a locality (Quarona) which is in Piedmont but the landrace was sampled in a different region probably deriving from recent exchanges made by amateurs which are highly susceptible of germplasm misidentification. Finally, this study examining popcorn landraces preserved *ex situ* is of particular importance for the conservation and safeguarding of these local genetic resources. Indeed, the *ex situ* cultivation of local popcorn varieties is essential for their preservation, as these resources have already faced near disappearance from the Italian territory. Relying solely on *in situ* conservation, mostly carried out by hobbyists and collectors, is not sufficiently reliable, as it can lead to admixture among local genotypes, thereby increasing the already present risk of genetic erosion and extinction.

Despite popcorn industry in Italy is very limited, the present work started to shed light on this neglected type of maize that, locally, has been poorly considered over the time. The information provided are relevant from the perspective of agricultural biodiversity conservation for future breeding and for the valorization of smallholder cultivation, mainly in marginal areas.

## Data Availability

The original contributions presented in the study are included in the article/**Supplementary Material**. Further inquiries can be directed to the corresponding author.
