# Supplementary figures and images for: Biodiversity of Northern Italy popcorn: a study on genetic diversity and agronomic performances of traditional landraces
Source: Front Plant Sci. 2025 Jun 13;16:1536714. doi: 10.3389/fpls.2025.1536714 (PMC12202408; doi:10.3389/fpls.2025.1536714)

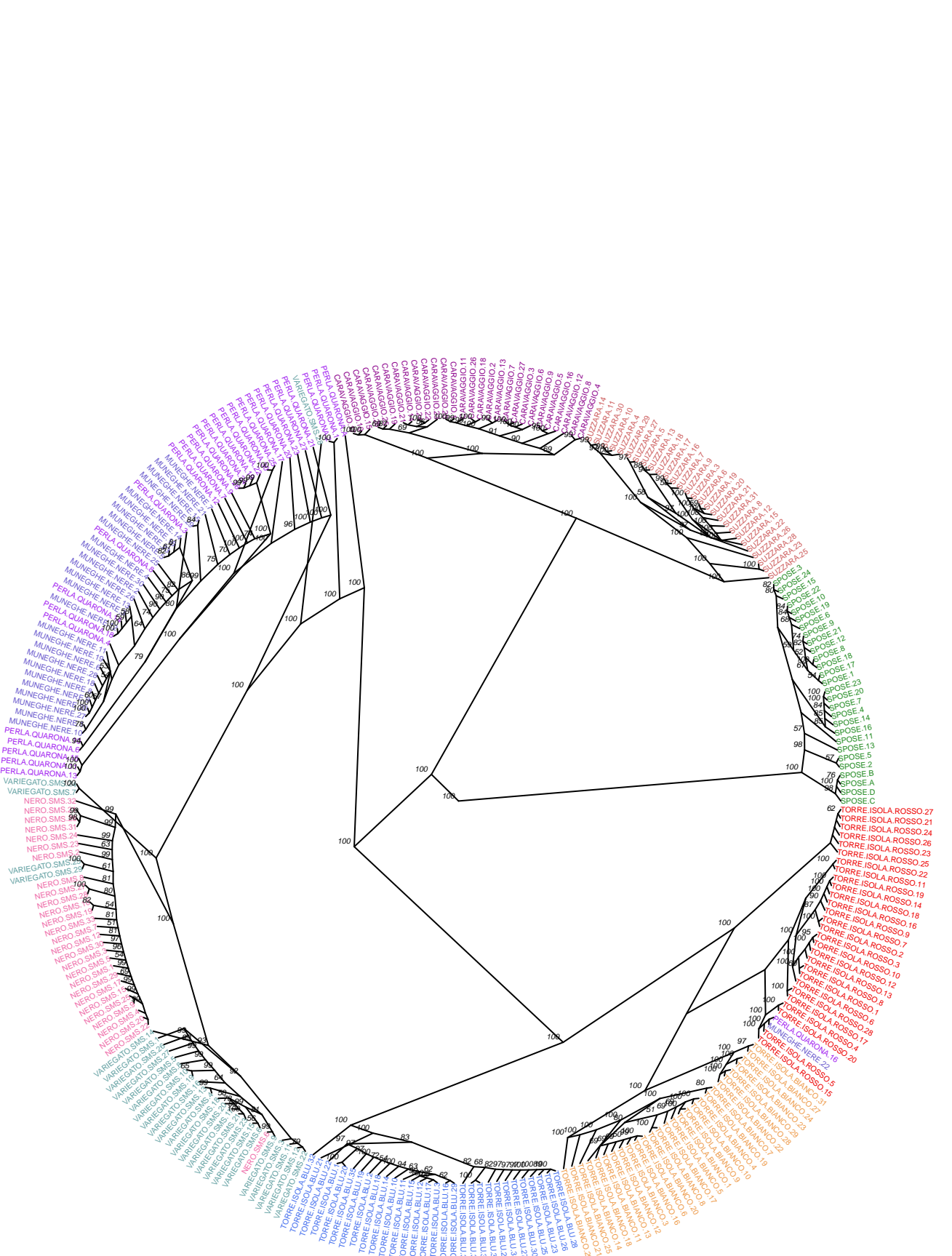

Supplement: Supplementary file 1 [file DataSheet1.pdf]
