# Supplementary material for: Biodiversity of Northern Italy popcorn: a study on genetic diversity and agronomic performances of traditional landraces
Source: Front Plant Sci. 2025 Jun 13;16:1536714. doi: 10.3389/fpls.2025.1536714 (PMC12202408; doi:10.3389/fpls.2025.1536714)
Supplement: Supplementary file 2 [file DataSheet2.docx]

Supplementary Material

# Supplementary Figures and Tables

## Supplementary Figures


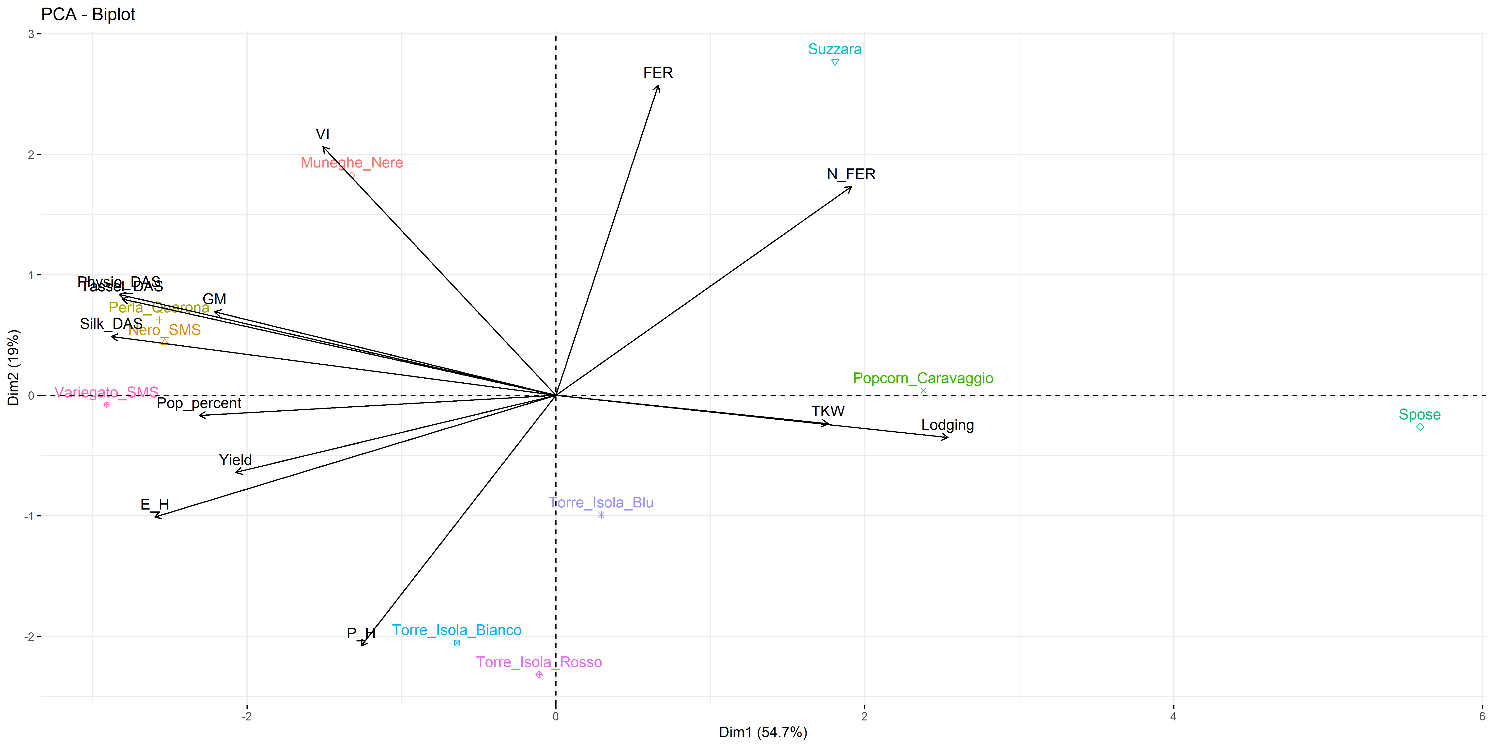


**Supplementary Figure S1.** Principal Component analysis of phenotypic traits in popcorn landraces.


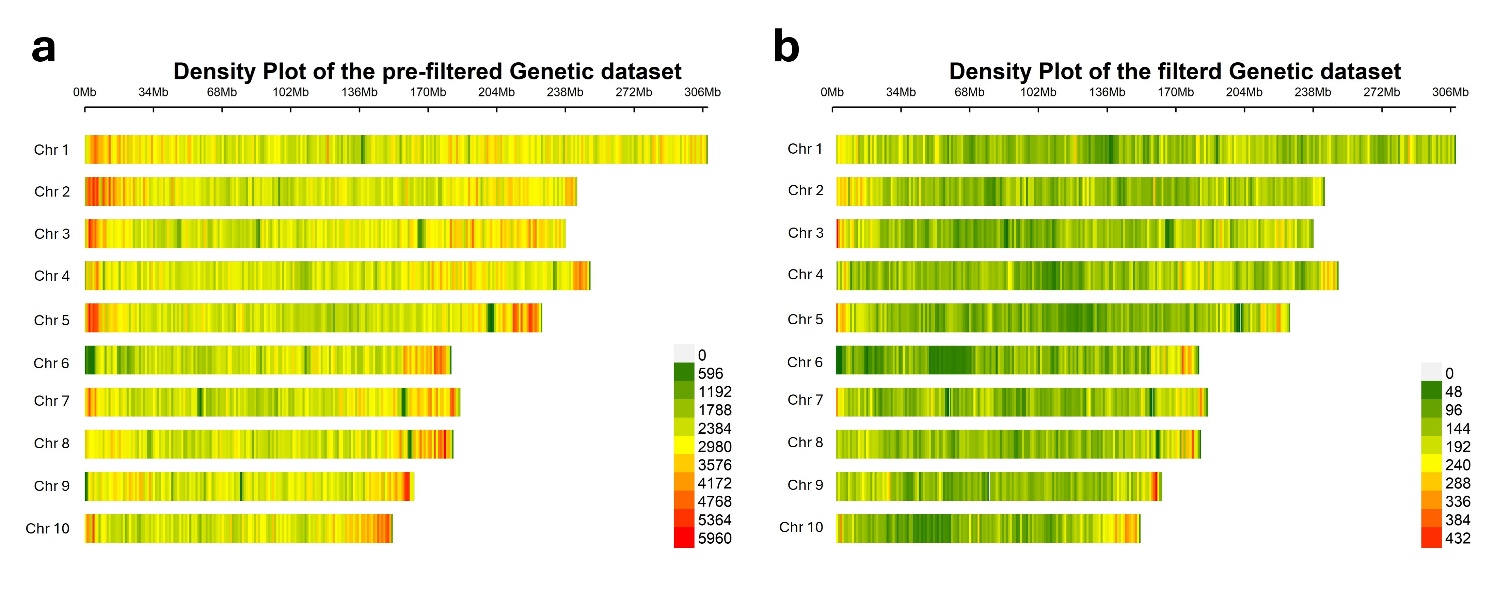


**Supplementary Figure S2.** Density plot of the (a) pre filtered genetic dataset containing 5,826,704 variants distributed along the maize chromosomes and (b) filtered genetic dataset containing 313,342 variants distributed along the maize chromosomes.

**Supplementary Figure S3.** Phylogenetic tree of the 282 individuals of the ten popcorn landraces evaluated.


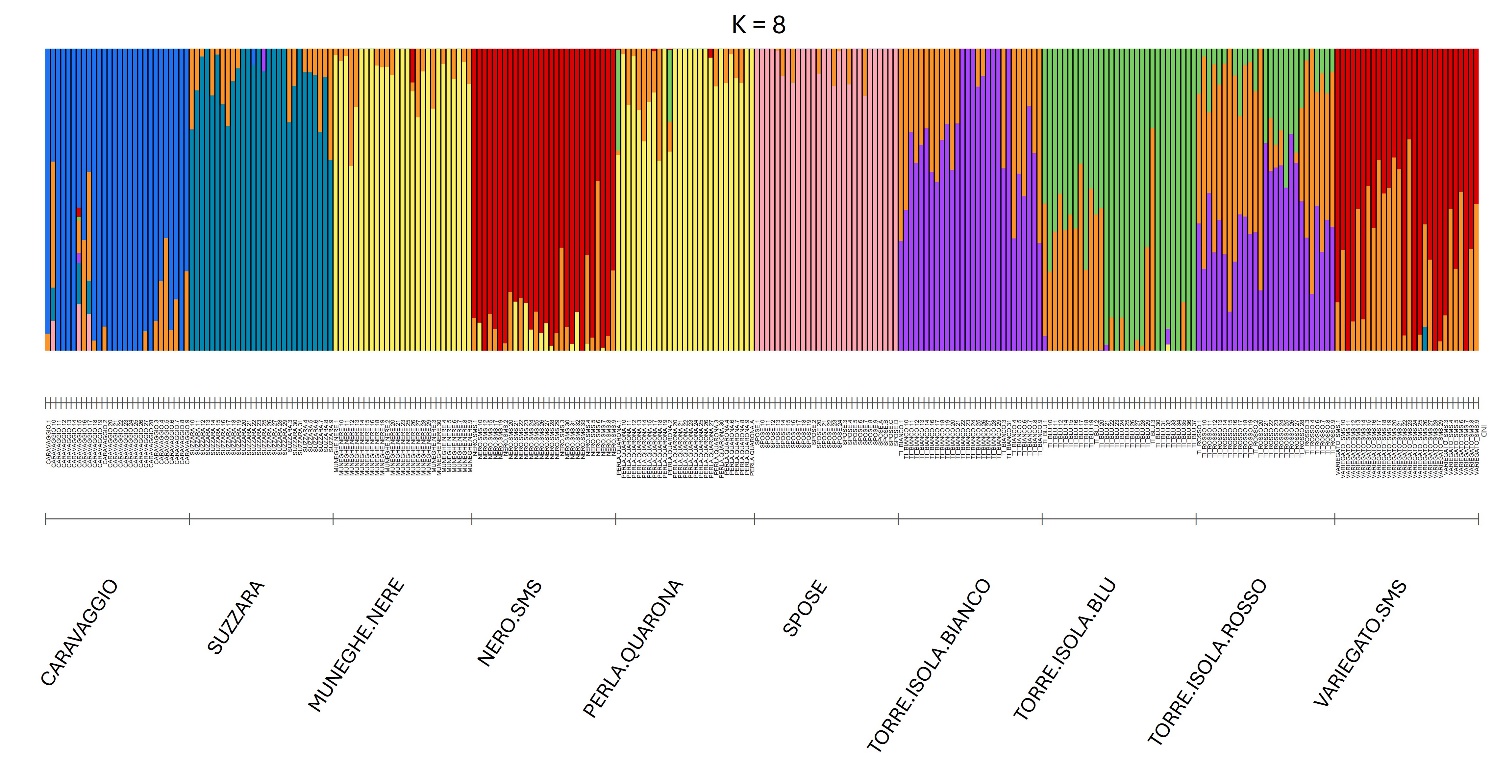


**Supplementary Figure S4.** Population genetic structure at K = 8 of the 282 individuals of the ten accessions evaluated in the present study. Different colors correspond to different ancestral populations.

**
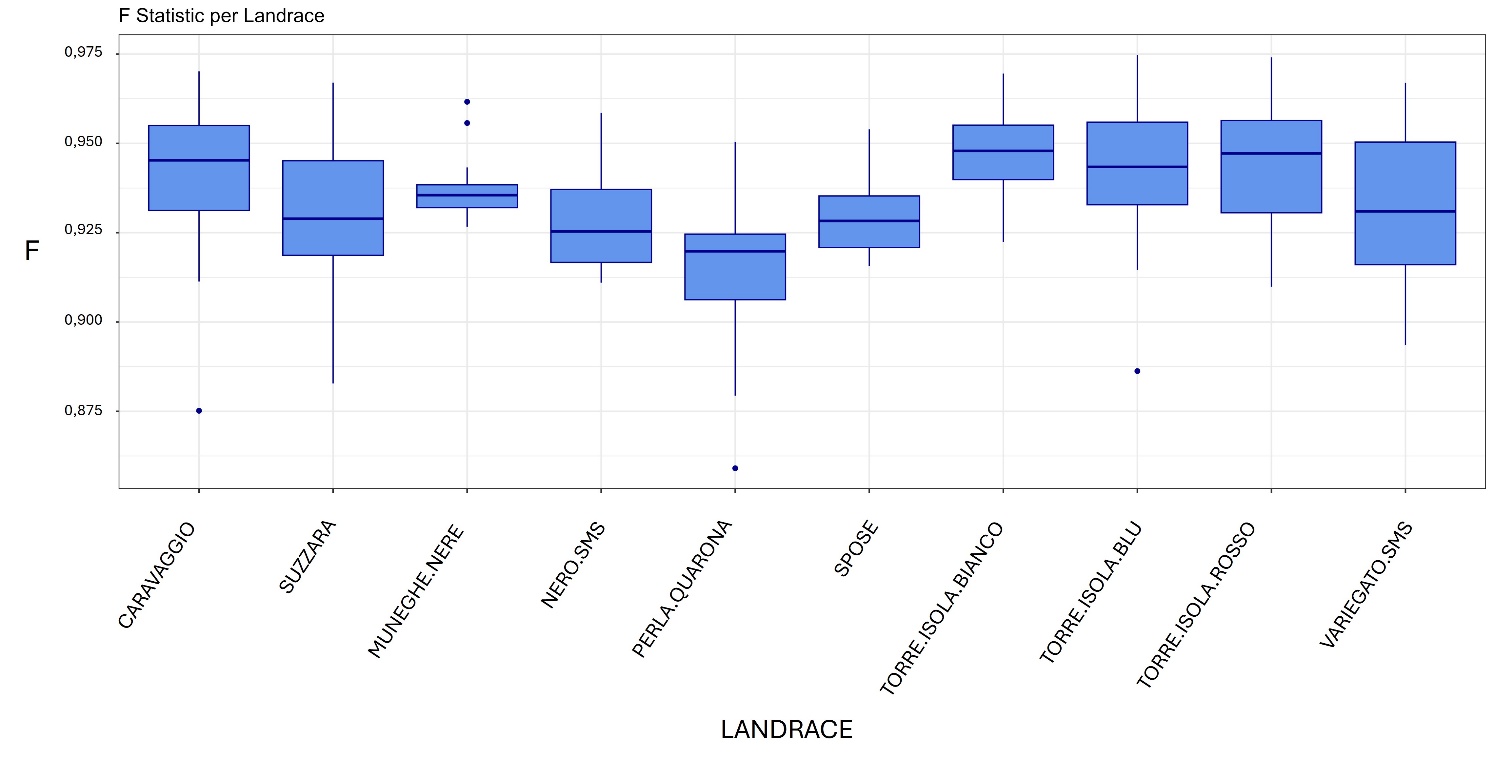
**

**Supplementary Figure S5.** Boxplot of the inbreeding coefficient (F) computed for each of the filtered SNPs and grouped by landrace.


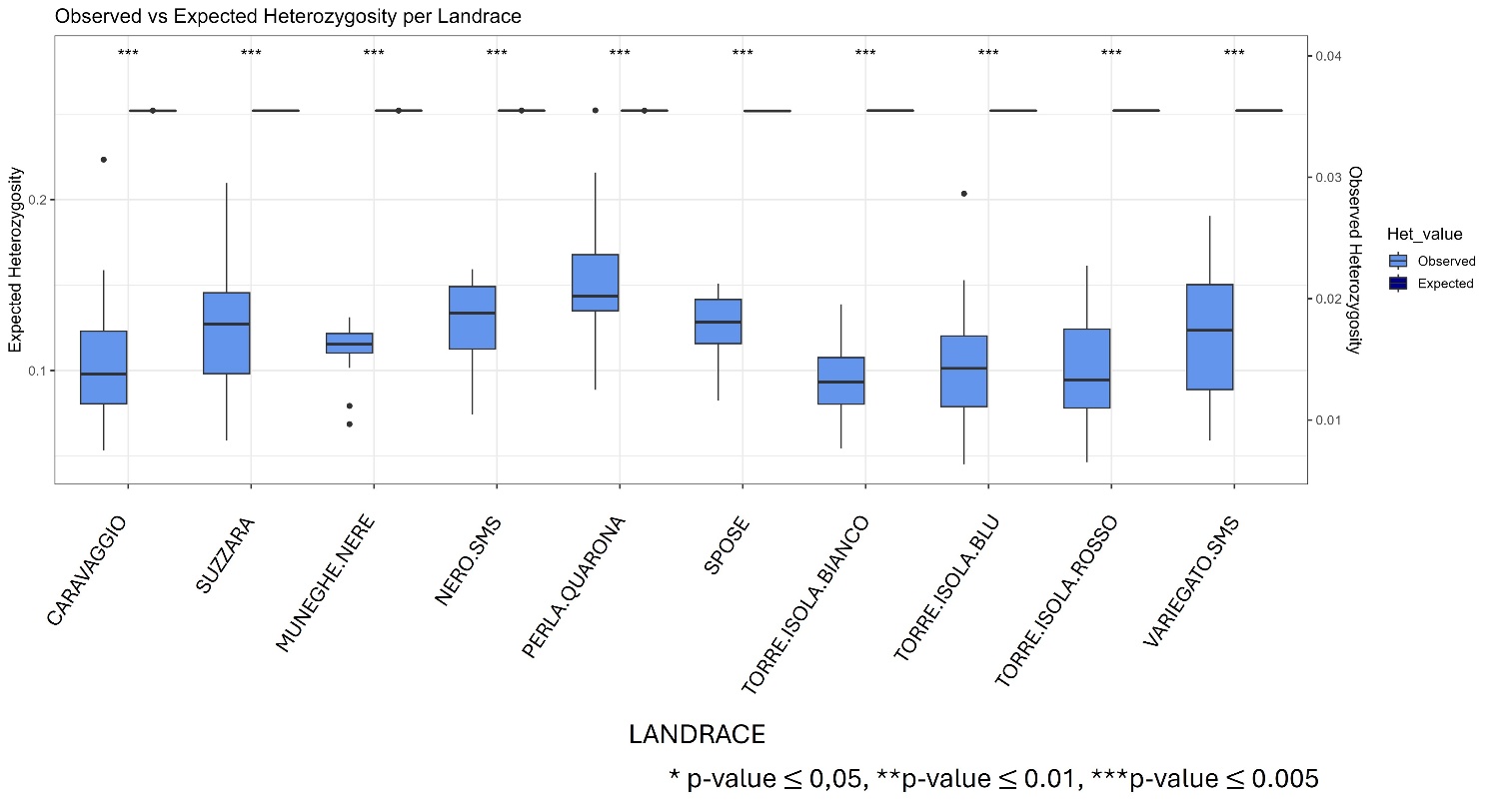


**Supplementary Figure S6.** Comparison between Expected and Observed heterozygosity for each popcorn landrace.


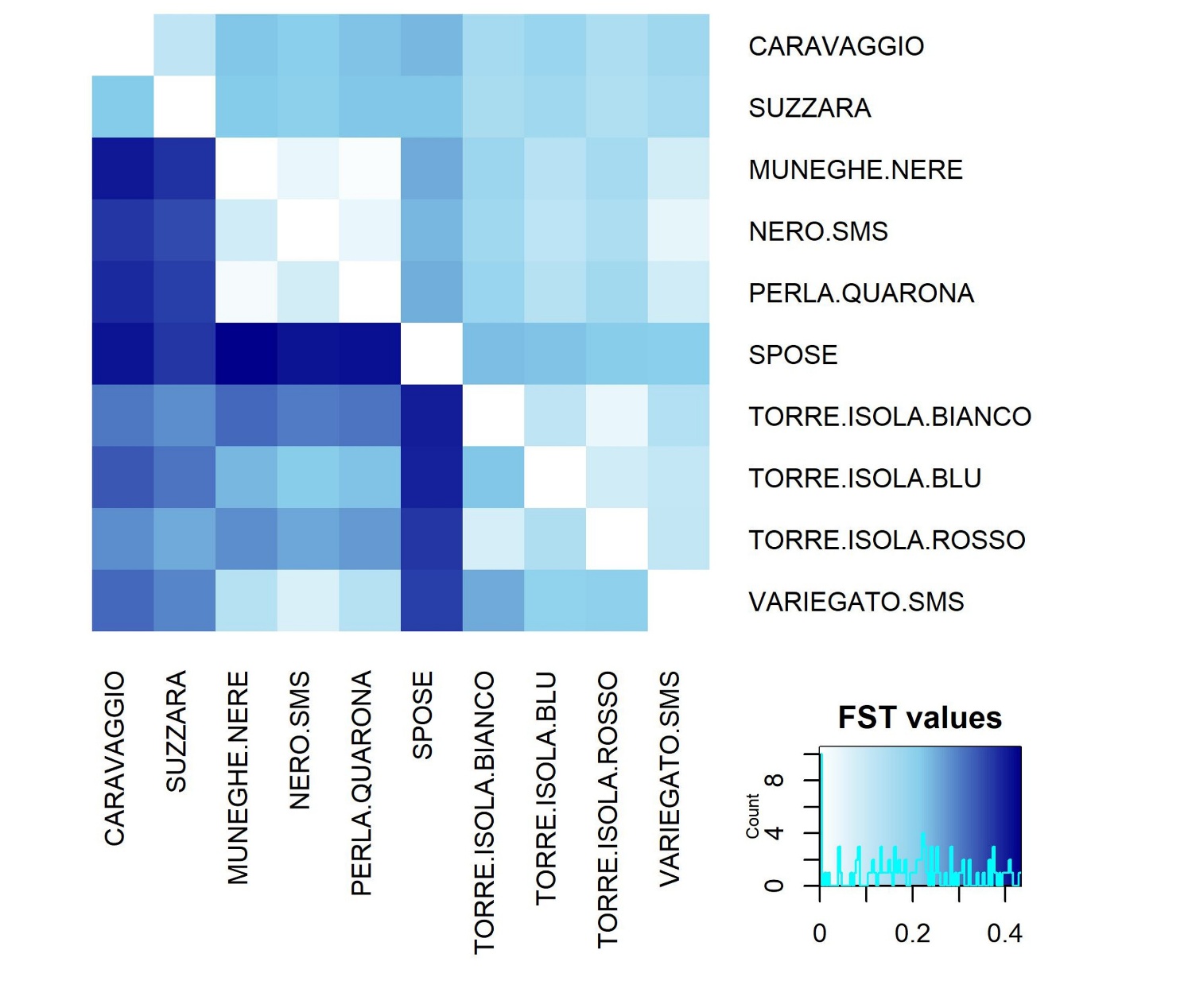


**Supplementary Figure S7.** Heatmap showing genetic differentiation based on FST values. Above the diagonal is reported the Weir and Cockerham mean F_ST_ estimate, whereas below the diagonal is the Weir and Cockerham weighted F_ST_ estimate.

## Supplementary Tables

**Table S1**: Detailed information regarding the ten Italian popcorn landraces characterized in the present study.

| **Accession name** | **Sampling site** | **Sampling year** | **Donor** |
| --- | --- | --- | --- |
| Popcorn Nero di San Martino Siccomario | San Martino Siccomario (PV) | 2018 | Francesco Slanzi |
| Popcorn Variegato di San Martino Siccomario | San Martino Siccomario (PV) | 2018 | Francesco Slanzi |
| Popcorn di Caravaggio | Caravaggio (BG) | 2020 | Giuseppe Cozzi |
| Popcorn Torre d'Isola Bianco | Torre d'Isola (PV) | 2018 | Carlo Boiocchi - Cascina Scaldasole |
| Popcorn Torre d'Isola Blu | Torre d'Isola (PV) | 2018 | Carlo Boiocchi - Cascina Scaldasole |
| Popcorn Torre d'Isola Rosso | Torre d'Isola (PV) | 2018 | Carlo Boiocchi - Cascina Scaldasole |
| Muneghe Nere | Seren del Grappa (BL) | 2018 | Tiziano Fantinel - Ass. Coltivare condividendo |
| Spose del Primiero | Imèr (TN) | 2019 | Simone Gaio |
| Perla di Quarona | Isola Dovarese (CR) | 2015 | Ass. Coltivare condividendo |
| Popcorn di Suzzara (MN) | Vespolate (NO) | 2019 | Giampietro Mossini |

**Table S2**: UPOV card with the phenotypic characters of popcorn landrace “Torre d’Isola Bianco”

| TORRE D'ISOLA BIANCO | | | | | | | | | | | | |
| --- | --- | --- | --- | --- | --- | --- | --- | --- | --- | --- | --- | --- |
| UPOV | CPVO TP/2/3 | CHARACTER | MEASURE | EVALUATION | | | | | | | | |
| 1 | 1 | First leaf: anthocyanin coloration of sheath |  |  |  | 3 |  | 5 |  |  |  |  |
| 2 | 2 | First leaf: apex shape |  |  | 2 |  |  |  |  |  |  |  |
| 3 | 3 | Leaves: green intensity |  |  | 2 | 3 |  |  |  |  |  |  |
| 5 | 4 | Leaf: angle between leaf and stem | degrees 25 |  |  | 3 |  |  |  |  |  |  |
| 6 | 5 | Leaf: attitude of leaf |  |  |  |  |  |  |  |  |  |  |
| 8 | 6 | Tassel: time of male flowering | DAS 83 |  |  |  |  |  |  |  |  |  |
| 9 | 7 | Tassel: anthocyanin coloration of ring of the glume |  | 1 |  |  |  |  |  |  |  |  |
| 10 | 8 | Tassel: anthocyanin coloration of the glumes |  |  |  |  |  |  |  | 7 |  |  |
| 11 | 9 | Tassel: anthocyanin coloration of the anthers |  |  |  |  |  | 5 |  |  |  |  |
| 12 | 10 | Tassel: angle between main axis and lateral branches | degrees 50 |  |  |  |  | 5 |  |  |  |  |
| 13 | 11 | Tassel: attitude of lateral branches |  |  |  |  |  | 5 |  | 7 |  |  |
| 14 | 12 | Tassel: number of lateral branches | n° 19-25 |  |  |  |  |  |  |  |  | 9 |
| 15 | 13 | Ear: silking time | DAS 86 |  |  |  |  |  |  |  |  |  |
| 16 | 14 | Ear: anthocyanin coloration of the silks |  |  |  | 3 |  |  |  | 7 |  |  |
| 17 | 15 | Stem: anthocyanin coloration of secondary roots |  |  |  | 3 |  | 5 |  |  |  |  |
| 18 | 16 | Tassel: density of main axis |  |  |  |  |  | 5 |  | 7 |  |  |
| 19 | 17 | Leaf: anthocyanin coloration of sheat |  | 1 |  |  |  |  |  |  |  |  |
| 20 | 18 | Stem: anthocyanin pigmentation of internodes |  | 1 |  | 3 |  |  |  |  |  |  |
| 21 | 19 | Tassel: length of main axis above lowest side branch |  |  |  |  |  | 5 |  |  |  |  |
| 22 | 20 | Tassel: length of main axis above highest side branch |  |  |  | 3 |  | 5 |  |  |  |  |
| 23 | 21 | Tassel: length of lowest lateral branch |  | 1 |  | 3 |  | 5 |  |  |  |  |
| 24.1 | 22.1 | Plant: height (tassel included) | cm 211 |  |  |  |  |  |  |  |  |  |
| 24.2.1 | # | Plant: ear height (upper-ear) | cm 128 |  |  |  |  |  |  |  |  |  |
| 25 | 23 | Plant: height of ear relative to plant length | 61% |  |  |  |  |  |  |  |  | 9 |
| 26 | 24 | Leaf: width of blade (leaf of the highest ear) |  | 1 |  |  |  |  |  |  |  |  |
| 27 | 25 | Ear: length of peduncle |  | 1 |  |  |  |  |  |  |  |  |
| 28 | 26 | Ear: length of ear | cm 11.1 | 1 |  |  |  |  |  |  |  |  |
| 29 | 27 | Ear: diameter of ear (midway) | mm 27.82 | 1 |  |  |  |  |  |  |  |  |
| 30 | 28 | Ear: shape of ear |  |  |  |  |  | 5 |  |  |  |  |
| 31 | 29 | Ear: number of rows |  |  |  |  |  | 5 |  | 7 |  |  |
| 32 | 30 | Ear: number of colors of graisn (only sweet or waxy) |  |  |  |  |  |  |  |  |  |  |
| 33 | 31 | Grain: intensity of yellow (only sweet) |  |  |  |  |  |  |  |  |  |  |
| 34 | 32 | Grain: lenght of yellow (only sweet) |  |  |  |  |  |  |  |  |  |  |
| 35 | 33 | Grain: width of yellow (only sweet) |  |  |  |  |  |  |  |  |  |  |
| 36 | 34 | Ear: type of grain |  |  |  |  |  |  |  | 7 |  |  |
| 37 | 35 | Ear: narrowing of the upper part of thegrain (only sweet) |  |  |  |  |  |  |  |  |  |  |
| 38 | 36 | Ear: color of the tip of grain |  |  | 2 |  |  |  |  |  |  |  |
| 39 | 37 | Ear: color of the dorsal side of grain |  |  |  |  |  |  |  |  |  |  |
| 40 | 38 | Shape of popped kernel (only pop-corn) | White | 1 |  |  |  |  |  |  |  |  |
| 41 | 39 | Ear: anthocyanin pigmentation of the glumes of cob |  |  |  |  |  | 5 |  |  |  |  |
|  |  | Physiological maturity | DAS 127 |  |  |  |  |  |  |  |  |  |

**Table S3**: UPOV card with the phenotypic characters of popcorn landrace “Torre d’Isola Blu”

| TORRE D'ISOLA BLU | | | | | | | | | | | | |
| --- | --- | --- | --- | --- | --- | --- | --- | --- | --- | --- | --- | --- |
| UPOV | CPVO TP/2/3 | CHARACTER | MEASURE | EVALUATION | | | | | | | | |
| 1 | 1 | First leaf: anthocyanin coloration of sheath |  |  |  |  |  |  |  |  |  |  |
| 2 | 2 | First leaf: apex shape |  |  |  |  |  |  |  |  |  |  |
| 3 | 3 | Leaves: green intensity |  |  | 2 |  |  |  |  |  |  |  |
| 5 | 4 | Leaf: angle between leaf and stem | degrees 25 |  |  | 3 |  |  |  |  |  |  |
| 6 | 5 | Leaf: attitude of leaf |  |  |  | 3 |  |  |  |  |  |  |
| 8 | 6 | Tassel: time of male flowering | DAS 84 |  |  |  |  |  |  |  |  |  |
| 9 | 7 | Tassel: anthocyanin coloration of ring of the glume |  | 1 |  |  |  |  |  |  |  |  |
| 10 | 8 | Tassel: anthocyanin coloration of the glumes |  |  |  | 3 |  |  |  |  |  |  |
| 11 | 9 | Tassel: anthocyanin coloration of the anthers |  |  |  | 3 |  |  |  |  |  |  |
| 12 | 10 | Tassel: angle between main axis and lateral branches | degrees |  |  |  |  |  |  | 7 |  |  |
| 13 | 11 | Tassel: attitude of lateral branches |  |  |  |  |  | 5 |  | 7 |  | 9 |
| 14 | 12 | Tassel: number of lateral branches |  |  |  |  |  |  |  |  |  | 9 |
| 15 | 13 | Ear: silking time | DAS 88 |  |  |  |  |  |  |  |  |  |
| 16 | 14 | Ear: anthocyanin coloration of the silks |  | 1 |  |  |  |  |  |  |  |  |
| 17 | 15 | Stem: anthocyanin coloration of secondary roots |  | 1 |  |  |  |  |  |  |  |  |
| 18 | 16 | Tassel: density of main axis |  |  |  |  |  | 5 |  | 7 |  |  |
| 19 | 17 | Leaf: anthocyanin coloration of sheat |  | 1 |  |  |  |  |  |  |  |  |
| 20 | 18 | Stem: anthocyanin pigmentation of internodes |  | 1 |  |  |  |  |  |  |  |  |
| 21 | 19 | Tassel: length of main axis above lowest side branch |  |  |  | 3 |  | 5 |  |  |  |  |
| 22 | 20 | Tassel: length of main axis above highest side branch |  |  |  | 3 |  | 5 |  |  |  |  |
| 23 | 21 | Tassel: length of lowest lateral branch |  |  |  | 3 |  | 5 |  |  |  |  |
| 24.1 | 22.1 | Plant: height (tassel included) | cm 183 |  |  |  |  |  |  |  |  |  |
| 24.2.1 | # | Plant: ear height (upper-ear) | cm 99 |  |  |  |  |  |  |  |  |  |
| 25 | 23 | Plant: height of ear relative to plant length | 54% |  |  |  |  |  |  | 7 |  |  |
| 26 | 24 | Leaf: width of blade (leaf of the highest ear) |  | 1 |  |  |  |  |  |  |  |  |
| 27 | 25 | Ear: length of peduncle |  | 1 |  |  |  |  |  |  |  |  |
| 28 | 26 | Ear: length of ear | cm 7.9 | 1 |  |  |  |  |  |  |  |  |
| 29 | 27 | Ear: diameter of ear (midway) | mm 24.75 | 1 |  |  |  |  |  |  |  |  |
| 30 | 28 | Ear: shape of ear |  |  |  |  |  |  |  | 7 |  |  |
| 31 | 29 | Ear: number of rows | n° 16-18 |  |  |  |  | 5 |  | 7 |  |  |
| 32 | 30 | Ear: number of colors of graisn (only sweet or waxy) |  |  |  |  |  |  |  |  |  |  |
| 33 | 31 | Grain: intensity of yellow (only sweet) |  |  |  |  |  |  |  |  |  |  |
| 34 | 32 | Grain: lenght of yellow (only sweet) |  |  |  |  |  |  |  |  |  |  |
| 35 | 33 | Grain: width of yellow (only sweet) |  |  |  |  |  |  |  |  |  |  |
| 36 | 34 | Ear: type of grain |  |  |  |  |  |  |  | 7 |  |  |
| 37 | 35 | Ear: narrowing of the upper part of thegrain (only sweet) |  |  |  |  |  |  |  |  |  |  |
| 38 | 36 | Ear: color of the tip of grain |  |  |  |  |  |  |  |  |  | 9 |
| 39 | 37 | Ear: color of the dorsal side of grain |  |  |  |  |  |  |  |  |  | 9 |
| 40 | 38 | Shape of popped kernel (only pop-corn) | White | 1 |  |  |  |  |  |  |  |  |
| 41 | 39 | Ear: anthocyanin pigmentation of the glumes of cob |  |  |  | 3 |  |  |  |  |  |  |
|  |  | Physiological maturity | DAS 129 |  |  |  |  |  |  |  |  |  |

**Table S4**: UPOV card with the phenotypic characters of popcorn landrace “Torre d’Isola Rosso”

| TORRE D'ISOLA ROSSO | | | | | | | | | | | | |
| --- | --- | --- | --- | --- | --- | --- | --- | --- | --- | --- | --- | --- |
| UPOV | CPVO TP/2/3 | CHARACTER | MEASURE | EVALUATION | | | | | | | | |
| 1 | 1 | First leaf: anthocyanin coloration of sheath |  |  |  | 3 |  | 5 |  |  |  |  |
| 2 | 2 | First leaf: apex shape |  |  | 2 |  |  |  |  |  |  |  |
| 3 | 3 | Leaves: green intensity |  |  | 2 | 3 |  |  |  |  |  |  |
| 5 | 4 | Leaf: angle between leaf and stem | degrees 25-30 |  |  | 3 |  |  |  |  |  |  |
| 6 | 5 | Leaf: attitude of leaf |  |  |  |  |  |  |  | 7 |  |  |
| 8 | 6 | Tassel: time of male flowering | DAS 79 |  |  |  |  |  |  |  |  |  |
| 9 | 7 | Tassel: anthocyanin coloration of ring of the glume |  | 1 |  |  |  |  |  |  |  |  |
| 10 | 8 | Tassel: anthocyanin coloration of the glumes |  | 1 |  | 3 |  |  |  |  |  |  |
| 11 | 9 | Tassel: anthocyanin coloration of the anthers |  |  |  |  |  |  |  |  |  |  |
| 12 | 10 | Tassel: angle between main axis and lateral branches |  |  |  |  |  |  |  | 7 |  |  |
| 13 | 11 | Tassel: attitude of lateral branches |  |  |  |  |  | 5 |  | 7 |  |  |
| 14 | 12 | Tassel: number of lateral branches | n° 20 |  |  |  |  |  |  | 7 |  | 9 |
| 15 | 13 | Ear: silking time | DAS 82 |  |  |  |  |  |  |  |  |  |
| 16 | 14 | Ear: anthocyanin coloration of the silks |  | 1 |  | 3 |  |  |  |  |  |  |
| 17 | 15 | Stem: anthocyanin coloration of secondary roots |  |  |  | 3 |  |  |  |  |  |  |
| 18 | 16 | Tassel: density of main axis |  |  |  |  |  | 5 |  | 7 |  |  |
| 19 | 17 | Leaf: anthocyanin coloration of sheat |  | 1 |  |  |  |  |  |  |  |  |
| 20 | 18 | Stem: anthocyanin pigmentation of internodes |  | 1 |  | 3 |  |  |  |  |  |  |
| 21 | 19 | Tassel: length of main axis above lowest side branch |  |  |  |  |  | 5 |  |  |  |  |
| 22 | 20 | Tassel: length of main axis above highest side branch |  |  |  |  |  | 5 |  |  |  |  |
| 23 | 21 | Tassel: length of lowest lateral branch |  |  |  | 3 |  | 5 |  | 7 |  |  |
| 24.1 | 22.1 | Plant: height (tassel included) | cm 201 |  |  |  |  |  |  |  |  |  |
| 24.2.1 | # | Plant: ear height (upper-ear) | cm 117 |  |  |  |  |  |  |  |  |  |
| 25 | 23 | Plant: height of ear relative to plant length | 58% |  |  |  |  |  |  |  |  | 9 |
| 26 | 24 | Leaf: width of blade (leaf of the highest ear) |  | 1 |  |  |  |  |  |  |  |  |
| 27 | 25 | Ear: length of peduncle |  | 1 |  |  |  |  |  |  |  |  |
| 28 | 26 | Ear: length of ear | cm 12.7 | 1 |  |  |  |  |  |  |  |  |
| 29 | 27 | Ear: diameter of ear (midway) | mm 27.84 | 1 |  |  |  |  |  |  |  |  |
| 30 | 28 | Ear: shape of ear |  |  |  |  |  | 5 |  |  |  |  |
| 31 | 29 | Ear: number of rows |  |  |  |  |  | 5 |  |  |  |  |
| 32 | 30 | Ear: number of colors of graisn (only sweet or waxy) |  |  |  |  |  |  |  |  |  |  |
| 33 | 31 | Grain: intensity of yellow (only sweet) |  |  |  |  |  |  |  |  |  |  |
| 34 | 32 | Grain: lenght of yellow (only sweet) |  |  |  |  |  |  |  |  |  |  |
| 35 | 33 | Grain: width of yellow (only sweet) |  |  |  |  |  |  |  |  |  |  |
| 36 | 34 | Ear: type of grain |  |  |  |  |  |  |  | 7 |  |  |
| 37 | 35 | Ear: narrowing of the upper part of thegrain (only sweet) |  |  |  |  |  |  |  |  |  |  |
| 38 | 36 | Ear: color of the tip of grain |  |  |  |  |  |  |  |  | 8 | 9 |
| 39 | 37 | Ear: color of the dorsal side of grain |  |  |  |  |  |  |  |  |  |  |
| 40 | 38 | Shape of popped kernel (only pop-corn) | White | 1 |  |  |  |  |  |  |  |  |
| 41 | 39 | Ear: anthocyanin pigmentation of the glumes of cob |  |  |  | 3 |  | 5 |  |  |  |  |
|  |  | Physiological maturity | DAS 125 |  |  |  |  |  |  |  |  |  |

**Table S5**: UPOV card with the phenotypic characters of popcorn landrace “Perla di Quarona”

| PERLA DI QUARONA | | | | | | | | | | | | |
| --- | --- | --- | --- | --- | --- | --- | --- | --- | --- | --- | --- | --- |
| UPOV | CPVO TP/2/3 | CHARACTER | MEASURE | EVALUATION | | | | | | | | |
| 1 | 1 | First leaf: anthocyanin coloration of sheath |  | 1 |  | 3 |  |  |  |  |  |  |
| 2 | 2 | First leaf: apex shape |  |  | 2 |  |  |  |  |  |  |  |
| 3 | 3 | Leaves: green intensity |  |  | 2 |  |  |  |  |  |  |  |
| 5 | 4 | Leaf: angle between leaf and stem | degrees 50 |  |  | 3 |  | 5 |  |  |  |  |
| 6 | 5 | Leaf: attitude of leaf |  |  |  |  |  |  |  | 7 |  |  |
| 8 | 6 | Tassel: time of male flowering | DAS 98 |  |  |  |  |  |  |  |  |  |
| 9 | 7 | Tassel: anthocyanin coloration of ring of the glume |  | 1 |  | 3 |  |  |  |  |  |  |
| 10 | 8 | Tassel: anthocyanin coloration of the glumes |  | 1 |  | 3 |  |  |  |  |  |  |
| 11 | 9 | Tassel: anthocyanin coloration of the anthers |  | 1 |  |  |  |  |  |  |  |  |
| 12 | 10 | Tassel: angle between main axis and lateral branches |  | 1 |  | 3 |  |  |  |  |  |  |
| 13 | 11 | Tassel: attitude of lateral branches |  |  |  |  |  | 5 |  | 7 |  |  |
| 14 | 12 | Tassel: number of lateral branches | n° 4-10 |  |  | 3 |  | 5 |  |  |  |  |
| 15 | 13 | Ear: silking time | DAS 99 |  |  |  |  |  |  |  |  |  |
| 16 | 14 | Ear: anthocyanin coloration of the silks |  | 1 |  |  |  |  |  |  |  |  |
| 17 | 15 | Stem: anthocyanin coloration of secondary roots |  |  |  | 3 |  |  |  |  |  |  |
| 18 | 16 | Tassel: density of main axis |  |  |  |  |  |  |  | 7 |  |  |
| 19 | 17 | Leaf: anthocyanin coloration of sheat |  | 1 |  |  |  |  |  |  |  |  |
| 20 | 18 | Stem: anthocyanin pigmentation of internodes |  | 1 |  |  |  |  |  |  |  |  |
| 21 | 19 | Tassel: length of main axis above lowest side branch |  |  |  | 3 |  |  |  |  |  |  |
| 22 | 20 | Tassel: length of main axis above highest side branch |  |  |  | 3 |  | 5 |  |  |  |  |
| 23 | 21 | Tassel: length of lowest lateral branch |  | 1 |  | 3 |  |  |  |  |  |  |
| 24.1 | 22.1 | Plant: height (tassel included) | cm 179 |  |  |  |  |  |  |  |  |  |
| 24.2.1 | # | Plant: ear height (upper-ear) | cm 131 |  |  |  |  |  |  |  |  |  |
| 25 | 23 | Plant: height of ear relative to plant length | 74% |  |  |  |  |  |  |  |  | 9 |
| 26 | 24 | Leaf: width of blade (leaf of the highest ear) |  |  |  |  |  |  |  |  |  |  |
| 27 | 25 | Ear: length of peduncle |  | 1 |  |  |  |  |  |  |  |  |
| 28 | 26 | Ear: length of ear | cm 9.2 | 1 |  |  |  |  |  |  |  |  |
| 29 | 27 | Ear: diameter of ear (midway) | mm 24.2 | 1 |  |  |  |  |  |  |  |  |
| 30 | 28 | Ear: shape of ear |  |  | 2 |  |  |  |  |  |  |  |
| 31 | 29 | Ear: number of rows | n° 12-14 |  |  |  |  | 5 |  |  |  |  |
| 32 | 30 | Ear: number of colors of graisn (only sweet or waxy) |  |  |  |  |  |  |  |  |  |  |
| 33 | 31 | Grain: intensity of yellow (only sweet) |  |  |  |  |  |  |  |  |  |  |
| 34 | 32 | Grain: lenght of yellow (only sweet) |  |  |  |  |  |  |  |  |  |  |
| 35 | 33 | Grain: width of yellow (only sweet) |  |  |  |  |  |  |  |  |  |  |
| 36 | 34 | Ear: type of grain |  | 1 |  |  |  |  |  | 7 |  |  |
| 37 | 35 | Ear: narrowing of the upper part of thegrain (only sweet) |  |  |  |  |  |  |  |  |  |  |
| 38 | 36 | Ear: color of the tip of grain |  |  |  |  |  |  |  |  |  | 9 |
| 39 | 37 | Ear: color of the dorsal side of grain |  |  |  |  |  |  |  |  |  |  |
| 40 | 38 | Shape of popped kernel (only pop-corn) | White | 1 | 2 |  |  |  |  |  |  |  |
| 41 | 39 | Ear: anthocyanin pigmentation of the glumes of cob |  | 1 |  |  |  |  |  |  |  |  |
|  |  | Physiological maturity | DAS 138 |  |  |  |  |  |  |  |  |  |

**Table S6**: UPOV card with the phenotypic characters of popcorn landrace “Nero di San Martino Siccomario”

| NERO DI SAN MARTINO SICCOMARIO | | | | | | | | | | | | |
| --- | --- | --- | --- | --- | --- | --- | --- | --- | --- | --- | --- | --- |
| UPOV | CPVO TP/2/3 | CHARACTER | MEASURE | EVALUATION | | | | | | | | |
| 1 | 1 | First leaf: anthocyanin coloration of sheath |  |  |  | 3 |  | 5 |  |  |  |  |
| 2 | 2 | First leaf: apex shape |  |  | 2 |  |  |  |  |  |  |  |
| 3 | 3 | Leaves: green intensity |  |  | 2 |  |  |  |  |  |  |  |
| 5 | 4 | Leaf: angle between leaf and stem | degrees 40-50 |  |  |  |  | 5 |  |  |  |  |
| 6 | 5 | Leaf: attitude of leaf |  |  |  |  |  | 5 |  | 7 |  |  |
| 8 | 6 | Tassel: time of male flowering | DAS 97 |  |  |  |  |  |  |  |  |  |
| 9 | 7 | Tassel: anthocyanin coloration of ring of the glume |  | 1 |  |  |  |  |  |  |  |  |
| 10 | 8 | Tassel: anthocyanin coloration of the glumes |  | 1 |  | 3 |  |  |  |  |  |  |
| 11 | 9 | Tassel: anthocyanin coloration of the anthers |  | 1 |  |  |  |  |  |  |  |  |
| 12 | 10 | Tassel: angle between main axis and lateral branches |  |  |  |  |  | 5 |  | 7 |  |  |
| 13 | 11 | Tassel: attitude of lateral branches |  |  |  |  |  |  |  | 7 |  | 9 |
| 14 | 12 | Tassel: number of lateral branches |  |  |  |  |  | 5 |  | 7 |  |  |
| 15 | 13 | Ear: silking time | DAS 99 |  |  |  |  |  |  |  |  |  |
| 16 | 14 | Ear: anthocyanin coloration of the silks |  | 1 |  |  |  |  |  |  |  |  |
| 17 | 15 | Stem: anthocyanin coloration of secondary roots |  |  |  | 3 |  | 5 |  |  |  |  |
| 18 | 16 | Tassel: density of main axis |  |  |  |  |  | 5 |  | 7 |  |  |
| 19 | 17 | Leaf: anthocyanin coloration of sheat |  | 1 |  |  |  |  |  |  |  |  |
| 20 | 18 | Stem: anthocyanin pigmentation of internodes |  | 1 |  |  |  |  |  |  |  |  |
| 21 | 19 | Tassel: length of main axis above lowest side branch |  |  |  | 3 |  | 5 |  |  |  |  |
| 22 | 20 | Tassel: length of main axis above highest side branch |  |  |  |  |  | 5 |  |  |  |  |
| 23 | 21 | Tassel: length of lowest lateral branch |  | 1 |  | 3 |  |  |  |  |  |  |
| 24.1 | 22.1 | Plant: height (tassel included) | cm 205 |  |  |  |  |  |  |  |  |  |
| 24.2.1 | # | Plant: ear height (upper-ear) | cm 137 |  |  |  |  |  |  |  |  |  |
| 25 | 23 | Plant: height of ear relative to plant length | 67% |  |  |  |  |  |  |  |  | 9 |
| 26 | 24 | Leaf: width of blade (leaf of the highest ear) |  | 1 |  | 3 |  |  |  |  |  |  |
| 27 | 25 | Ear: length of peduncle |  | 1 |  |  |  |  |  |  |  |  |
| 28 | 26 | Ear: length of ear | cm 7.9 | 1 |  |  |  |  |  |  |  |  |
| 29 | 27 | Ear: diameter of ear (midway) | mm 24.99 | 1 |  |  |  |  |  |  |  |  |
| 30 | 28 | Ear: shape of ear |  |  |  |  |  |  |  | 7 |  |  |
| 31 | 29 | Ear: number of rows | n° 12.14 |  |  | 3 |  | 5 |  |  |  |  |
| 32 | 30 | Ear: number of colors of graisn (only sweet or waxy) |  |  |  |  |  |  |  |  |  |  |
| 33 | 31 | Grain: intensity of yellow (only sweet) |  |  |  |  |  |  |  |  |  |  |
| 34 | 32 | Grain: lenght of yellow (only sweet) |  |  |  |  |  |  |  |  |  |  |
| 35 | 33 | Grain: width of yellow (only sweet) |  |  |  |  |  |  |  |  |  |  |
| 36 | 34 | Ear: type of grain |  |  |  |  |  |  |  | 7 |  |  |
| 37 | 35 | Ear: narrowing of the upper part of thegrain (only sweet) |  |  |  |  |  |  |  |  |  |  |
| 38 | 36 | Ear: color of the tip of grain |  |  |  |  |  |  |  |  |  |  |
| 39 | 37 | Ear: color of the dorsal side of grain |  |  |  |  |  |  |  |  |  |  |
| 40 | 38 | Shape of popped kernel (only pop-corn) | White | 1 |  | 3 |  |  |  |  |  |  |
| 41 | 39 | Ear: anthocyanin pigmentation of the glumes of cob |  | 1 |  |  |  |  |  |  |  |  |
|  |  | Physiological maturity | 140 |  |  |  |  |  |  |  |  |  |

**Table S7**: UPOV card with the phenotypic characters of popcorn landrace “Variegato di San Martino Siccomario”

| VARIEGATO DI SAN MARTINO SICCOMARIO | | | | | | | | | | | | |
| --- | --- | --- | --- | --- | --- | --- | --- | --- | --- | --- | --- | --- |
| UPOV | CPVO TP/2/3 | CHARACTER | MEASURE | EVALUATION | | | | | | | | |
| 1 | 1 | First leaf: anthocyanin coloration of sheath |  |  |  | 3 |  | 5 |  |  |  |  |
| 2 | 2 | First leaf: apex shape |  |  | 2 |  |  |  |  |  |  |  |
| 3 | 3 | Leaves: green intensity |  |  | 2 | 3 |  |  |  |  |  |  |
| 5 | 4 | Leaf: angle between leaf and stem | degrees 30-50 |  |  |  |  | 5 |  |  |  |  |
| 6 | 5 | Leaf: attitude of leaf |  |  |  |  |  | 5 |  |  |  |  |
| 8 | 6 | Tassel: time of male flowering | DAS 98 |  |  |  |  |  |  |  |  |  |
| 9 | 7 | Tassel: anthocyanin coloration of ring of the glume |  | 1 |  |  |  |  |  |  |  |  |
| 10 | 8 | Tassel: anthocyanin coloration of the glumes |  | 1 |  | 3 |  |  |  |  |  |  |
| 11 | 9 | Tassel: anthocyanin coloration of the anthers |  | 1 |  |  |  |  |  |  |  |  |
| 12 | 10 | Tassel: angle between main axis and lateral branches |  |  |  |  |  |  |  | 7 |  | 9 |
| 13 | 11 | Tassel: attitude of lateral branches |  |  |  |  |  | 5 |  | 7 |  | 9 |
| 14 | 12 | Tassel: number of lateral branches |  |  |  |  |  | 5 |  | 7 |  |  |
| 15 | 13 | Ear: silking time | DAS 100 |  |  |  |  |  |  |  |  |  |
| 16 | 14 | Ear: anthocyanin coloration of the silks |  | 1 |  |  |  |  |  |  |  |  |
| 17 | 15 | Stem: anthocyanin coloration of secondary roots |  |  |  |  |  | 5 |  | 7 |  |  |
| 18 | 16 | Tassel: density of main axis |  |  |  |  |  | 5 |  |  |  |  |
| 19 | 17 | Leaf: anthocyanin coloration of sheat |  | 1 |  |  |  |  |  |  |  |  |
| 20 | 18 | Stem: anthocyanin pigmentation of internodes |  | 1 |  |  |  |  |  |  |  |  |
| 21 | 19 | Tassel: length of main axis above lowest side branch |  |  |  | 3 |  | 5 |  |  |  |  |
| 22 | 20 | Tassel: length of main axis above highest side branch |  |  |  |  |  | 5 |  |  |  |  |
| 23 | 21 | Tassel: length of lowest lateral branch |  |  |  | 3 |  | 5 |  | 7 |  |  |
| 24.1 | 22.1 | Plant: height (tassel included) | cm 206 |  |  |  |  |  |  |  |  |  |
| 24.2.1 | # | Plant: ear height (upper-ear) | cm 133 |  |  |  |  |  |  |  |  |  |
| 25 | 23 | Plant: height of ear relative to plant length | 64% |  |  |  |  |  |  |  |  | 9 |
| 26 | 24 | Leaf: width of blade (leaf of the highest ear) |  |  |  | 3 |  | 5 |  |  |  |  |
| 27 | 25 | Ear: length of peduncle |  | 1 |  |  |  |  |  |  |  |  |
| 28 | 26 | Ear: length of ear | cm 9.8 | 1 |  |  |  |  |  |  |  |  |
| 29 | 27 | Ear: diameter of ear (midway) | mm 26.38 | 1 |  |  |  |  |  |  |  |  |
| 30 | 28 | Ear: shape of ear |  |  |  |  |  |  |  | 7 |  |  |
| 31 | 29 | Ear: number of rows |  |  |  |  |  | 5 |  |  |  |  |
| 32 | 30 | Ear: number of colors of graisn (only sweet or waxy) |  |  |  |  |  |  |  |  |  |  |
| 33 | 31 | Grain: intensity of yellow (only sweet) |  |  |  |  |  |  |  |  |  |  |
| 34 | 32 | Grain: lenght of yellow (only sweet) |  |  |  |  |  |  |  |  |  |  |
| 35 | 33 | Grain: width of yellow (only sweet) |  |  |  |  |  |  |  |  |  |  |
| 36 | 34 | Ear: type of grain |  |  |  |  |  |  |  | 7 |  |  |
| 37 | 35 | Ear: narrowing of the upper part of thegrain (only sweet) |  |  |  |  |  |  |  |  |  |  |
| 38 | 36 | Ear: color of the tip of grain |  |  | 2 |  |  |  |  |  |  | 9 |
| 39 | 37 | Ear: color of the dorsal side of grain |  |  |  |  |  |  |  |  |  |  |
| 40 | 38 | Shape of popped kernel (only pop-corn) | White | 1 |  |  |  |  |  |  |  |  |
| 41 | 39 | Ear: anthocyanin pigmentation of the glumes of cob |  | 1 |  |  |  |  |  |  |  |  |
|  |  | Physiological maturity | DAS 100 |  |  |  |  |  |  |  |  |  |

**Table S8**: UPOV card with the phenotypic characters of popcorn landrace “Suzzara”

| SUZZARA | | | | | | | | | | | | |
| --- | --- | --- | --- | --- | --- | --- | --- | --- | --- | --- | --- | --- |
| UPOV | CPVO TP/2/3 | CHARACTER | MEASURE | EVALUATION | | | | | | | | |
| 1 | 1 | First leaf: anthocyanin coloration of sheath |  | 1 |  |  |  |  |  |  |  |  |
| 2 | 2 | First leaf: apex shape |  |  |  | 3 |  |  |  |  |  |  |
| 3 | 3 | Leaves: green intensity |  |  | 2 |  |  |  |  |  |  |  |
| 5 | 4 | Leaf: angle between leaf and stem | degrees 50 |  |  |  |  | 5 |  |  |  |  |
| 6 | 5 | Leaf: attitude of leaf |  |  |  |  |  | 5 |  |  |  |  |
| 8 | 6 | Tassel: time of male flowering | DAS 80 |  |  |  |  |  |  |  |  |  |
| 9 | 7 | Tassel: anthocyanin coloration of ring of the glume |  |  |  |  |  | 5 |  |  |  |  |
| 10 | 8 | Tassel: anthocyanin coloration of the glumes |  | 1 |  |  |  |  |  |  |  |  |
| 11 | 9 | Tassel: anthocyanin coloration of the anthers |  | 1 |  |  |  |  |  |  |  |  |
| 12 | 10 | Tassel: angle between main axis and lateral branches | degrees 35-40 |  |  | 3 |  |  |  |  |  |  |
| 13 | 11 | Tassel: attitude of lateral branches |  |  |  |  |  | 5 |  |  |  |  |
| 14 | 12 | Tassel: number of lateral branches |  |  |  |  |  | 5 |  | 7 |  |  |
| 15 | 13 | Ear: silking time | DAS 82 |  |  |  |  |  |  |  |  |  |
| 16 | 14 | Ear: anthocyanin coloration of the silks |  | 1 |  |  |  |  |  |  |  |  |
| 17 | 15 | Stem: anthocyanin coloration of secondary roots |  | 1 |  |  |  |  |  |  |  |  |
| 18 | 16 | Tassel: density of main axis |  |  |  |  |  | 5 |  |  |  |  |
| 19 | 17 | Leaf: anthocyanin coloration of sheat |  | 1 |  |  |  |  |  |  |  |  |
| 20 | 18 | Stem: anthocyanin pigmentation of internodes |  | 1 |  |  |  |  |  |  |  |  |
| 21 | 19 | Tassel: length of main axis above lowest side branch |  |  |  |  |  | 5 |  |  |  |  |
| 22 | 20 | Tassel: length of main axis above highest side branch |  |  |  |  |  | 5 |  |  |  |  |
| 23 | 21 | Tassel: length of lowest lateral branch |  |  |  | 3 |  |  |  |  |  |  |
| 24.1 | 22.1 | Plant: height (tassel included) | cm 161 | 1 |  | 3 |  |  |  |  |  |  |
| 24.2.1 | # | Plant: ear height (upper-ear) | cm 78 |  |  |  |  |  |  |  |  |  |
| 25 | 23 | Plant: height of ear relative to plant length | 48% |  |  |  |  | 5 |  |  |  |  |
| 26 | 24 | Leaf: width of blade (leaf of the highest ear) |  | 1 |  |  |  |  |  |  |  |  |
| 27 | 25 | Ear: length of peduncle |  |  |  |  |  |  |  | 7 |  |  |
| 28 | 26 | Ear: length of ear | cm 11.6 | 1 |  |  |  |  |  |  |  |  |
| 29 | 27 | Ear: diameter of ear (midway) | mm 19.3 | 1 |  |  |  |  |  |  |  |  |
| 30 | 28 | Ear: shape of ear |  |  | 2 |  |  |  |  |  |  |  |
| 31 | 29 | Ear: number of rows | n° 12-14 |  |  | 3 |  |  |  |  |  |  |
| 32 | 30 | Ear: number of colors of graisn (only sweet or waxy) |  |  |  |  |  |  |  |  |  |  |
| 33 | 31 | Grain: intensity of yellow (only sweet) |  |  |  |  |  |  |  |  |  |  |
| 34 | 32 | Grain: lenght of yellow (only sweet) |  |  |  |  |  |  |  |  |  |  |
| 35 | 33 | Grain: width of yellow (only sweet) |  |  |  |  |  |  |  |  |  |  |
| 36 | 34 | Ear: type of grain |  |  | 2 |  |  |  |  | 7 |  |  |
| 37 | 35 | Ear: narrowing of the upper part of thegrain (only sweet) |  |  |  |  |  |  |  |  |  |  |
| 38 | 36 | Ear: color of the tip of grain |  |  |  | 3 |  |  |  |  |  |  |
| 39 | 37 | Ear: color of the dorsal side of grain |  |  |  |  |  |  |  |  |  |  |
| 40 | 38 | Shape of popped kernel (only pop-corn) | Cream | 1 |  |  |  |  |  |  |  |  |
| 41 | 39 | Ear: anthocyanin pigmentation of the glumes of cob |  | 1 |  |  |  |  |  |  |  |  |
|  |  | Physiological maturity | DAS 127 |  |  |  |  |  |  |  |  |  |

**Table S9**: UPOV card with the phenotypic characters of popcorn landrace “Caravaggio”

| CARAVAGGIO | | | | | | | | | | | | |
| --- | --- | --- | --- | --- | --- | --- | --- | --- | --- | --- | --- | --- |
| UPOV | CPVO TP/2/3 | CHARACTER | MEASURE | EVALUATION | | | | | | | | |
| 1 | 1 | First leaf: anthocyanin coloration of sheath |  | 1 |  |  |  |  |  |  |  |  |
| 2 | 2 | First leaf: apex shape |  |  | 2 |  |  |  |  |  |  |  |
| 3 | 3 | Leaves: green intensity |  |  | 2 |  |  |  |  |  |  |  |
| 5 | 4 | Leaf: angle between leaf and stem |  |  |  |  |  | 5 |  | 7 |  |  |
| 6 | 5 | Leaf: attitude of leaf |  |  |  |  |  | 5 |  | 7 |  |  |
| 8 | 6 | Tassel: time of male flowering | DAS 75 |  |  |  |  |  |  |  |  |  |
| 9 | 7 | Tassel: anthocyanin coloration of ring of the glume |  | 1 |  | 3 |  |  |  |  |  |  |
| 10 | 8 | Tassel: anthocyanin coloration of the glumes |  | 1 |  |  |  |  |  |  |  |  |
| 11 | 9 | Tassel: anthocyanin coloration of the anthers |  | 1 |  |  |  |  |  |  |  |  |
| 12 | 10 | Tassel: angle between main axis and lateral branches |  |  |  | 3 |  |  |  |  |  |  |
| 13 | 11 | Tassel: attitude of lateral branches |  |  |  |  |  |  |  | 7 |  | 9 |
| 14 | 12 | Tassel: number of lateral branches |  |  |  |  |  |  |  | 7 |  | 9 |
| 15 | 13 | Ear: silking time | DAS 76 |  |  |  |  |  |  |  |  |  |
| 16 | 14 | Ear: anthocyanin coloration of the silks |  | 1 |  |  |  |  |  |  |  |  |
| 17 | 15 | Stem: anthocyanin coloration of secondary roots |  | 1 |  | 3 |  |  |  |  |  |  |
| 18 | 16 | Tassel: density of main axis |  |  |  |  |  | 5 |  | 7 |  |  |
| 19 | 17 | Leaf: anthocyanin coloration of sheat |  | 1 |  |  |  |  |  |  |  |  |
| 20 | 18 | Stem: anthocyanin pigmentation of internodes |  | 1 |  |  |  |  |  |  |  |  |
| 21 | 19 | Tassel: length of main axis above lowest side branch |  |  |  |  |  | 5 |  |  |  |  |
| 22 | 20 | Tassel: length of main axis above highest side branch |  |  |  |  |  | 5 |  |  |  |  |
| 23 | 21 | Tassel: length of lowest lateral branch |  |  |  |  |  | 5 |  | 7 |  | 9 |
| 24.1 | 22.1 | Plant: height (tassel included) | cm 162 |  |  |  |  |  |  |  |  |  |
| 24.2.1 | # | Plant: ear height (upper-ear) | cm 76 |  |  |  |  |  |  |  |  |  |
| 25 | 23 | Plant: height of ear relative to plant length | 47% |  |  |  |  | 5 |  |  |  |  |
| 26 | 24 | Leaf: width of blade (leaf of the highest ear) |  | 1 |  |  |  |  |  |  |  |  |
| 27 | 25 | Ear: length of peduncle |  | 1 |  |  |  |  |  |  |  |  |
| 28 | 26 | Ear: length of ear | cm 13.6 | 1 |  |  |  |  |  |  |  |  |
| 29 | 27 | Ear: diameter of ear (midway) | mm 25.26 | 1 |  |  |  |  |  |  |  |  |
| 30 | 28 | Ear: shape of ear |  |  |  |  |  | 5 |  |  |  |  |
| 31 | 29 | Ear: number of rows | n° 12-16 |  |  | 3 |  | 5 |  | 7 |  |  |
| 32 | 30 | Ear: number of colors of graisn (only sweet or waxy) |  |  |  |  |  |  |  |  |  |  |
| 33 | 31 | Grain: intensity of yellow (only sweet) |  |  |  |  |  |  |  |  |  |  |
| 34 | 32 | Grain: lenght of yellow (only sweet) |  |  |  |  |  |  |  |  |  |  |
| 35 | 33 | Grain: width of yellow (only sweet) |  |  |  |  |  |  |  |  |  |  |
| 36 | 34 | Ear: type of grain |  |  |  |  |  |  |  | 7 |  |  |
| 37 | 35 | Ear: narrowing of the upper part of thegrain (only sweet) |  |  |  |  |  |  |  |  |  |  |
| 38 | 36 | Ear: color of the tip of grain |  |  |  |  | 4 |  |  |  |  |  |
| 39 | 37 | Ear: color of the dorsal side of grain |  |  |  |  |  |  |  |  |  |  |
| 40 | 38 | Shape of popped kernel (only pop-corn) | White-yellowish | 1 | 2 |  |  |  |  |  |  |  |
| 41 | 39 | Ear: anthocyanin pigmentation of the glumes of cob |  | 1 |  |  |  |  |  |  |  |  |
|  |  | Physiological maturity | DAS 121 |  |  |  |  |  |  |  |  |  |

**Table S10**: UPOV card with the phenotypic characters of popcorn landrace “Spose del Primiero”

| SPOSE DEL PRIMIERO | | | | | | | | | | | | |
| --- | --- | --- | --- | --- | --- | --- | --- | --- | --- | --- | --- | --- |
| UPOV | CPVO TP/2/3 | CHARACTER | MEASURE | EVALUATION | | | | | | | | |
| 1 | 1 | First leaf: anthocyanin coloration of sheath |  | 1 |  |  |  |  |  |  |  |  |
| 2 | 2 | First leaf: apex shape |  |  | 2 |  |  |  |  |  |  |  |
| 3 | 3 | Leaves: green intensity |  |  | 2 |  |  |  |  |  |  |  |
| 5 | 4 | Leaf: angle between leaf and stem | degrees 50 |  |  |  |  | 5 |  |  |  |  |
| 6 | 5 | Leaf: attitude of leaf |  |  |  |  |  | 5 |  |  |  |  |
| 8 | 6 | Tassel: time of male flowering | DAS 65 |  |  |  |  |  |  |  |  |  |
| 9 | 7 | Tassel: anthocyanin coloration of ring of the glume |  | 1 |  |  |  |  |  |  |  |  |
| 10 | 8 | Tassel: anthocyanin coloration of the glumes |  |  |  | 3 |  |  |  |  |  |  |
| 11 | 9 | Tassel: anthocyanin coloration of the anthers |  |  |  | 3 |  |  |  |  |  |  |
| 12 | 10 | Tassel: angle between main axis and lateral branches |  |  |  | 3 |  |  |  |  |  |  |
| 13 | 11 | Tassel: attitude of lateral branches |  |  |  | 3 |  |  |  |  |  |  |
| 14 | 12 | Tassel: number of lateral branches |  | 1 |  | 3 |  |  |  |  |  |  |
| 15 | 13 | Ear: silking time | DAS 67 |  |  |  |  | 5 |  | 7 |  |  |
| 16 | 14 | Ear: anthocyanin coloration of the silks |  | 1 |  |  |  |  |  |  |  |  |
| 17 | 15 | Stem: anthocyanin coloration of secondary roots |  | 1 |  |  |  |  |  |  |  |  |
| 18 | 16 | Tassel: density of main axis |  |  |  | 3 |  |  |  |  |  |  |
| 19 | 17 | Leaf: anthocyanin coloration of sheat |  |  |  |  |  | 5 |  |  |  |  |
| 20 | 18 | Stem: anthocyanin pigmentation of internodes |  |  |  | 3 |  |  |  |  |  |  |
| 21 | 19 | Tassel: length of main axis above lowest side branch |  |  |  |  |  |  |  |  |  | 9 |
| 22 | 20 | Tassel: length of main axis above highest side branch |  |  |  |  |  | 5 |  |  |  |  |
| 23 | 21 | Tassel: length of lowest lateral branch |  |  |  | 3 |  |  |  |  |  |  |
| 24.1 | 22.1 | Plant: height (tassel included) | cm 175 |  |  |  |  |  |  |  |  |  |
| 24.2.1 | # | Plant: ear height (upper-ear) | cm 75 |  |  |  |  |  |  |  |  |  |
| 25 | 23 | Plant: height of ear relative to plant length | 43% |  |  | 3 |  |  |  |  |  |  |
| 26 | 24 | Leaf: width of blade (leaf of the highest ear) |  | 1 |  |  |  |  |  |  |  |  |
| 27 | 25 | Ear: length of peduncle |  |  |  | 3 |  |  |  |  |  |  |
| 28 | 26 | Ear: length of ear | cm 11.8 | 1 |  |  |  |  |  |  |  |  |
| 29 | 27 | Ear: diameter of ear (midway) | mm 25 | 1 |  |  |  |  |  |  |  |  |
| 30 | 28 | Ear: shape of ear |  |  | 2 |  |  |  |  |  |  |  |
| 31 | 29 | Ear: number of rows | n° 14-18 |  |  |  |  | 5 |  |  |  |  |
| 32 | 30 | Ear: number of colors of graisn (only sweet or waxy) |  |  |  |  |  |  |  |  |  |  |
| 33 | 31 | Grain: intensity of yellow (only sweet) |  |  |  |  |  |  |  |  |  |  |
| 34 | 32 | Grain: lenght of yellow (only sweet) |  |  |  |  |  |  |  |  |  |  |
| 35 | 33 | Grain: width of yellow (only sweet) |  |  |  |  |  |  |  |  |  |  |
| 36 | 34 | Ear: type of grain |  | 1 |  |  |  |  |  | 7 |  |  |
| 37 | 35 | Ear: narrowing of the upper part of thegrain (only sweet) |  |  |  |  |  |  |  |  |  |  |
| 38 | 36 | Ear: color of the tip of grain |  |  | 2 |  |  |  |  |  |  |  |
| 39 | 37 | Ear: color of the dorsal side of grain |  |  |  |  |  |  |  |  |  |  |
| 40 | 38 | Shape of popped kernel (only pop-corn) |  |  |  |  |  |  |  |  |  |  |
| 41 | 39 | Ear: anthocyanin pigmentation of the glumes of cob |  | 1 |  |  |  |  |  |  |  |  |
|  |  | Physiological maturity | DAS 112 |  |  |  |  |  |  |  |  |  |

**Table S11**: UPOV card with the phenotypic characters of popcorn landrace “Muneghe Nere”

| MUNEGHE NERE | | | | | | | | | | | | |
| --- | --- | --- | --- | --- | --- | --- | --- | --- | --- | --- | --- | --- |
| UPOV | CPVO TP/2/3 | CHARACTER | MEASURE | EVALUATION | | | | | | | | |
| 1 | 1 | First leaf: anthocyanin coloration of sheath |  | 1 |  |  |  |  |  |  |  |  |
| 2 | 2 | First leaf: apex shape |  | 1 | 2 |  |  |  |  |  |  |  |
| 3 | 3 | Leaves: green intensity |  |  | 2 |  |  |  |  |  |  |  |
| 5 | 4 | Leaf: angle between leaf and stem | degrees 50-70 |  |  |  |  | 5 |  | 7 |  |  |
| 6 | 5 | Leaf: attitude of leaf |  |  |  |  |  | 5 |  | 7 |  |  |
| 8 | 6 | Tassel: time of male flowering | DAS 98 |  |  |  |  |  |  |  |  |  |
| 9 | 7 | Tassel: anthocyanin coloration of ring of the glume |  | 1 |  |  |  |  |  |  |  |  |
| 10 | 8 | Tassel: anthocyanin coloration of the glumes |  | 1 |  | 3 |  |  |  |  |  |  |
| 11 | 9 | Tassel: anthocyanin coloration of the anthers |  | 1 |  |  |  |  |  |  |  |  |
| 12 | 10 | Tassel: angle between main axis and lateral branches | degrees 15 | 1 |  |  |  |  |  |  |  |  |
| 13 | 11 | Tassel: attitude of lateral branches |  |  |  |  |  |  |  | 7 |  | 9 |
| 14 | 12 | Tassel: number of lateral branches | n° > 11 |  |  |  |  |  |  | 7 |  | 9 |
| 15 | 13 | Ear: silking time | DAS 92 |  |  |  |  |  |  |  |  |  |
| 16 | 14 | Ear: anthocyanin coloration of the silks |  | 1 |  |  |  |  |  |  |  |  |
| 17 | 15 | Stem: anthocyanin coloration of secondary roots |  |  |  | 3 |  |  |  |  |  |  |
| 18 | 16 | Tassel: density of main axis |  |  |  |  |  |  |  | 7 |  |  |
| 19 | 17 | Leaf: anthocyanin coloration of sheat |  | 1 |  |  |  |  |  |  |  |  |
| 20 | 18 | Stem: anthocyanin pigmentation of internodes |  | 1 |  |  |  |  |  |  |  |  |
| 21 | 19 | Tassel: length of main axis above lowest side branch |  |  |  | 3 |  |  |  |  |  |  |
| 22 | 20 | Tassel: length of main axis above highest side branch |  |  |  | 3 |  | 5 |  |  |  |  |
| 23 | 21 | Tassel: length of lowest lateral branch |  | 1 |  | 3 |  |  |  |  |  |  |
| 24.1 | 22.1 | Plant: height (tassel included) | cm 148 |  |  |  |  |  |  |  |  |  |
| 24.2.1 | # | Plant: ear height (upper-ear) | cm 99 |  |  |  |  |  |  |  |  |  |
| 25 | 23 | Plant: height of ear relative to plant length | 67% |  |  |  |  |  |  |  |  | 9 |
| 26 | 24 | Leaf: width of blade (leaf of the highest ear) |  | 1 |  |  |  |  |  |  |  |  |
| 27 | 25 | Ear: length of peduncle |  | 1 |  | 3 |  | 5 |  |  |  |  |
| 28 | 26 | Ear: length of ear | cm 9.6 | 1 |  |  |  |  |  |  |  |  |
| 29 | 27 | Ear: diameter of ear (midway) | mm 22.2 | 1 |  |  |  |  |  |  |  |  |
| 30 | 28 | Ear: shape of ear |  |  | 2 |  |  |  |  |  |  |  |
| 31 | 29 | Ear: number of rows | n° 12-14 |  |  |  |  | 5 |  |  |  |  |
| 32 | 30 | Ear: number of colors of graisn (only sweet or waxy) |  |  |  |  |  |  |  |  |  |  |
| 33 | 31 | Grain: intensity of yellow (only sweet) |  |  |  |  |  |  |  |  |  |  |
| 34 | 32 | Grain: lenght of yellow (only sweet) |  |  |  |  |  |  |  |  |  |  |
| 35 | 33 | Grain: width of yellow (only sweet) |  |  |  |  |  |  |  |  |  |  |
| 36 | 34 | Ear: type of grain |  |  |  |  |  |  |  | 7 |  |  |
| 37 | 35 | Ear: narrowing of the upper part of thegrain (only sweet) |  |  |  |  |  |  |  |  |  |  |
| 38 | 36 | Ear: color of the tip of grain |  |  |  |  |  |  |  |  |  | 9 |
| 39 | 37 | Ear: color of the dorsal side of grain |  |  |  |  |  |  |  |  |  |  |
| 40 | 38 | Shape of popped kernel (only pop-corn) | White | 1 | 2 |  |  |  |  |  |  |  |
| 41 | 39 | Ear: anthocyanin pigmentation of the glumes of cob |  | 1 |  |  |  |  |  |  |  |  |
|  |  | Physiological maturity | DAS 136 |  |  |  |  |  |  |  |  |  |
